# Supplementary material for: An Unsupervised Algorithm for Host Identification in Flaviviruses
Source: Life (Basel). 2021 May 14;11(5):442. doi: 10.3390/life11050442 (PMC8157105; doi:10.3390/life11050442)
Supplement: Supplementary file 1 [file life-11-00442-s001.zip › supplementary/5-Truong_MDPI_Supplementary_Tables_rev.pdf]

SUPPLEMENTARY MATERIAL

An unsupervised algorithm for host identification in flaviviruses

Phuoc Truong Nguyen [1, 2], Santiago Garcia-Vallve [3], Pere Puigbò [1, 4, 5, *]

[1] *Department of Biology, University of Turku, Turku, Finland.*

[2] *Currently at the Department of Virology, Medicum, Faculty of Medicine, University of Helsinki, Helsinki, Finland.*

[3] *Research Group in Cheminformatics & Nutrition, Department of Biochemistry and Biotechnology, Rovira i Virgili University, Tarragona, Catalonia, Spain*

[4] *Currently at the Department of Biochemistry and Biotechnology, Rovira i Virgili University, Tarragona, Catalonia, Spain*

[5] *Currently at the Nutrition and Health Unit, Eurecat Technology Centre of Catalonia, Reus, Catalonia, Spain*

[*] *Corresponding author: pepuav@utu.fi*

Supplementary table S1. List of flaviviruses (genus *Flavivirus*) and their putative hosts.

Virus name	Accession codes		GC3	Based on the literature		Correspondence Analysis (CA)			
	CDS	AA		Putative hosts (H) and vectors (V)	Classification	Dim. 1	Dim. 2	Centroid classification	The Nearest host
Aedes flavivirus	NC_012932.1	YP_003029843.1	0.531	*Aedes albopictus* (H) [1]<br>*Aedes flavopictus* (H) [1]	IOFV	0.395	-1.636	IOFV / Mosquito	*Aedes albopictus*
Alfuy virus	AY898809.1	AAX82481.1	0.525	*Mus musculus* (H) [1]<br>*Centropus phasianinus* (H) [1]<br>*Mammalia* (H) [2]<br>*Culex pullus* (V) [3]<br>*Culex sitiens* (V) [4]	MBFV	-0.025	-0.498	MBFV / Vertebrate, mosquito	*Columba livia*
Alkhurma hemorrhagic fever virus	NC_004355.1	NP_722551.1	0.579	*Ixodes petauristae* (V) [5]<br>*Ixodes ceylonensis* (V) [5]<br>*Homo sapiens* (H) [5, 6]	TBFV	0.792	0.745	TBFV / Tick, vertebrate	*Sus scrofa*
Anopheles flavivirus variant 1	NC_031327.1	YP_009305197.1	0.525	*Anopheles gambiae* (H) [1]	IOFV	0.208	-1.993	IOFV / Mosquito	*Aedes albopictus*
Anopheles flavivirus variant 2	KX148547.1	AOR51360.1	0.519	*Anopheles gambiae* (H) [2]	IOFV	-0.037	-1.419	IOFV / Mosquito, vertebrate	*Aedes albopictus*
Apoi virus	NC_003676.1	NP_620045.1	0.501	*Apodemus argenteus* (H) [1]	UVFV	-0.966	0.752	MBFV / Vertebrate	*Homo sapiens*
Bagaza virus	NC_012534.1	YP_002790883.1	0.529	*Culex tritaeniorhynchus* (V) [7]<br>*Homo sapiens* (H) [7]<br>*Alectoris rufa* (H) [8]<br>*Phasianus colchicus* (H) [8]	MBFV	-0.081	0.126	MBFV / Vertebrate, mosquito	*Gallus gallus*
Bainyik virus	KM225264.1	AIJ19433.1	0.528	*Culicidae* (V) [1]<br>*Aedes albopictus* (V) [1]<br>*Mus musculus* (H) [1]<br>*Aedes* sp. (V) [1]<br>Vertebrates (H) [9]	MBFV	-0.093	0.111	MBFV / Vertebrate, mosquito	*Gallus gallus*
Bamaga virus	NC_033725.1	YP_009345036.1	0.500	*Culex sitiens* (V) [1]<br>*Marsupialia* (H) [9]	MBFV	-1.003	0.537	UVFV / Vertebrate	*Alligator mississipiensis*
Banzi virus	DQ859056.1	ABI54472.1	0.548	*Culex rubinotus* (V) [10]<br>*Mansonia africana* (V) [10]<br>*Mesocricetus auratus* (H) [10]<br>*Mastomys natalensis* (H) [10]	MBFV	-0.191	1.293	MBFV / Vertebrate	*Myotis brandtii*
Bouboui virus	NC_033693.1	YP_009344961.1	0.493	*Antilocapra* (H) [1]<br>*Rodentia* (H) [1]<br>*Cercopithecus nictitans* (H) [1]<br>*Papio papio* (H) [1]<br>*Anopheles paludis* (V) [10]<br>*Eretmapodites inornatus* (V) [10]<br>*Aedes* spp. (V) [10]<br>*Culex* spp. (V) [10]	MBFV	-1.111	0.669	MBFV / Vertebrate	*Alligator mississipiensis*
Bussuquara virus	NC_009026.2	YP_001040004.1	0.521	*Chlorocebus aethiops* (H) [1]<br>*Homo sapiens* (H) [1]<br>*Proechimys* spp. (H) [11]<br>*Alouatta belzebul* (H) [12]	MBFV	-0.200	0.071	MBFV / Vertebrate, mosquito	*Gallus gallus*

Cacipacore virus	NC_026623.1	YP_009126874.1	0.519	*Formicarius analis* (H) [1]<br>*Homo sapiens* (H) [13]	MBFV	-0.224	-0.236	MBFV / Vertebrate, mosquito	*Columba livia*
Calbertado virus	KX669689.1	ASA45776.1	0.567	*Culex tarsalis* (H) [2]<br>*Culex pipiens* (H) [14]	IOFV	1.330	-2.695	IOFV / Mosquito	*Aedes albopictus*
Cell fusing agent virus	NC_001564.2	YP_009259257.1	0.566	*Aedes aegypti* (H) [1]<br>*Culicidae* (H) [15]	IOFV	0.678	0.253	MBFV / Mosquito, vertebrate, tick	*Bos taurus*
Chaoyang virus	NC_017086.1	YP_005454257.1	0.492	*Culicidae* (H) [1]	dhIOFV	-0.536	-1.048	dhIOFV / Vertebrate, mosquito	*Anas platyrhynchos*
Culex flavivirus	NC_008604.2	YP_899469.2	0.603	*Culex pipiens* (H) [1]	IOFV	1.874	-1.680	IOFV / Mosquito	*Culex quinquefasciatus*
Culiseta flavivirus	NC_030290.1	YP_009256193.1	0.492	*Culiseta melanura* (H) [1]	IOFV	-0.293	-2.071	IOFV / Mosquito	*Aedes aegypti*
Deer tick virus	AF311056.1	AAL32169.1	0.561	*Ixodes scapularis* [1]	TBFV	0.392	0.525	TBFV / Tick, vertebrate	*Bos taurus*
Dengue virus 1	NC_001477.1	NP_059433.1	0.462	*Aedes aegypti* (V) [1]<br>*Aedes albopictus* (V) [1]<br>*Homo sapiens* (H) [1]	MBFV	-1.244	-0.627	dhIOFV / Vertebrate	*Anas platyrhynchos*
Dengue virus 2	NC_001474.2	NP_056776.2	0.459	*Aedes aegypti* (V) [1]<br>*Erythrocebus patas* (H) [1]<br>*Homo sapiens* (H) [1]<br>*Aedes furcifer* (V) [1]<br>*Aedes taylori* (V) [1]	MBFV	-1.475	-0.059	dhIOFV / Vertebrate	*Anas platyrhynchos*
Dengue virus 3	NC_001475.2	YP_001621843.1	0.468	*Erythrocebus patas* (H) [1]<br>*Homo sapiens* (H) [1]<br>*Diceromyia* (V) [1]<br>*Aedimorphus* (V) [1]<br>*Stegomyia* (V) [1]	MBFV	-1.437	0.706	MBFV / Vertebrate	*Alligator mississipiensis*
Dengue virus 4	NC_002640.1	NP_073286.1	0.481	*Aedes aegypti* (V) [1]<br>*Aedes albopictus* (V) [1]<br>*Homo sapiens* (H) [1]<br>*Aedes polynesiensis* (V) [1]	MBFV	-1.176	1.138	MBFV / Vertebrate	*Homo sapiens*
Donggang virus	NC_016997.1	YP_005352889.1	0.484	*Culicidae* (V) [1]<br>*Aedes* sp. (V) [1]	dhIOFV	-1.092	-0.490	dhIOFV / Vertebrate	*Anas platyrhynchos*
Edge Hill virus	NC_030289.1	YP_009256192.1	0.488	*Macropodidae* (H) [1]<br>*Culex annulirostris* (V) [10]<br>*Anopheles meraukensis* (V) [10]<br>*Aedes vigilax* (V) [10]	MBFV	-1.091	0.123	UVFV / Vertebrate	*Alligator mississipiensis*
Entebbe bat virus	NC_008718.1	YP_950477.1	0.567	*Chiroptera* (H) [1]	MBFV	0.426	0.881	MBFV / Vertebrate, tick	*Myotis davidii*
Far Eastern tick-borne encephalitis virus	JX498940.1	AFV41132.1	0.589	*Ixodes persulcatus* (V) [16]<br>*Mus musculus* (H) [16]	TBFV	0.958	0.646	TBFV / Tick, vertebrate	*Sus scrofa*
Fitzroy River Virus	KM361634.1	AKH03452.1	0.483	*Aedes normanensis* (V) [2]<br>*Anopheles amictus* (V) [17]<br>*Culex annulirostris* (V) [17]<br>*Mammalia* (H) [17]<br>*Aves* (H) [17]	MBFV	-1.367	1.286	MBFV / Vertebrate	*Homo sapiens*

Gadgets Gully virus	NC_033723.1	YP_009345034.1	0.553	*Aves* (H) [1]<br>*Homo sapiens* (H) [1]<br>*Ixodes uriae* (V) [18]	TBFV	0.515	0.116	TBFV / Tick, vertebrate, mosquito	*Gallus gallus*
Hanko virus	NC_030401.1	YP_009259489.1	0.488	*Culicidae* (H) [1]<br>*Ochlerotatus punctor* (H) [19]<br>*Ochlerotatus caspius* (H) [19]	IOFV	-0.211	-2.423	IOFV / Mosquito	*Aedes aegypti*
Iguape virus	AY632538.4	AAV34154.1	0.557	Rodents (H) [20]<br>Sentinel mouse (H) [20]<br>Marsupials (H) [20]<br>Birds (H) [20]	MBFV	0.449	0.341	MBFV / Vertebrate, mosquito, tick	*Bos taurus*
Ilheus virus	NC_009028.2	YP_001040006.1	0.581	*Culex* (V) [1]<br>*Haemagogus* (V) [1]<br>*Psorophora* (V) [1]<br>*Aves* (H) [1]<br>*Homo sapiens* (H) [1]<br>*Sabethes* (V) [1]<br>*Ochlerotatus* (V) [1]<br>*Trichoprosopon* (V) [1]	MBFV	0.643	1.266	MBFV / Vertebrate, tick	*Sus scrofa*
Ilomantsi virus	NC_024805.1	YP_009056847.1	0.476	*Culicidae* (H) [1]	dhIOFV	-0.963	-1.410	dhIOFV / Vertebrate, mosquito	*Xenopus laevis*
Israel turkey meningoencephalomyelitis virus	KC734549.1	AGV15505.1	0.522	*Meleagris gallopavo* (H) [2]<br>*Ochlerotatus caspius* (V) [21]<br>*Culicoides imicola* (V) [21]<br>*Culex pipiens* (V) [21]<br>*Phlebotomus papatasi* (V) [21]<br>*Culicoides distinctipennis* (V) [22]	MBFV	-0.218	-0.028	MBFV / Vertebrate, mosquito	*Gallus gallus*
Japanese encephalitis virus	NC_001437.1	NP_059434.1	0.557	*Culex tritaeniorhynchus* (V) [1]<br>*Ardeidae* (H) [1]<br>*Homo sapiens* (H) [1]<br>*Equus caballus* (H) [1]<br>*Sus scrofa* (H) [1]<br>*Bos Taurus* (H) [1]<br>*Culex gelidus* (V) [1]	MBFV	0.544	0.060	MBFV / Vertebrate, mosquito, tick	*Gallus gallus*
Jugra virus	NC_033699.1	YP_009344969.1	0.491	*Cynopterus brachyotis* (H) [1]<br>*Aedes* sp. (V) [10]<br>*Uranotaenia* sp. (V) [10]	MBFV	-1.201	0.991	MBFV / Vertebrate	*Homo sapiens*
Jutiapa virus	NC_026620.1	YP_009126871.1	0.447	*Sigmodon hispidus* (H) [1]	UVFV	-1.797	-0.035	UVFV / Vertebrate	*Xenopus laevis*
Kadam virus	NC_033724.1	YP_009345035.1	0.560	*Homo sapiens* [1]<br>*Rhipicephalus pravus* (V) [23]<br>*Rhipicephalus pulchellus* (V) [24]<br>*Amblyomma variegatum* (V) [24]<br>*Hyalomma dromedariil* (V) [25]<br>*Dermacentor variabilis* (V) [26]<br>*Mus musculus* (H) [26]	TBFV	0.298	0.923	TBFV / Tick, vertebrate	*Myotis davidii*
Kamiti River virus	NC_005064.1	NP_891560.1	0.541	*Aedes* (H) [1]	IOFV	0.248	-0.732	dhIOFV / Mosquito, vertebrate	*Aedes albopictus*
Karshi virus	NC_006947.1	YP_224133.1	0.608	*Homo sapiens* (H) [1]<br>*Rodentia* (H) [1]<br>*Ornithodoros papillipes* (V) [27]<br>*Mus musculus* (H) [27]	TBFV	1.376	1.387	TBFV / Tick, vertebrate	*Sus scrofa*

Kedougou virus	NC_012533.1	YP_002790882.1	0.595	*Culicidae* (V) [1]<br>*Aedes dalzieli* (V) [28]<br>*Homo sapiens* (H) [29]	MBFV	0.816	1.715	TBFV / Vertebrate	*Sus scrofa*
Kokobera virus	NC_009029.2	YP_001040007.1	0.527	*Aedes albopictus* (V) [1]<br>*Macropus* (H) [1]<br>*Wallabia* (H) [1]<br>*Homo sapiens* (H) [1]<br>*Culex annulirostris* (V) [1]<br>*Ochlerotatus vigilax* (V) [1]<br>*Ochlerotatus camptorhynchus* (V) [1]<br>*Culex sitiens* (V) [30]	MBFV	0.036	0.089	MBFV / Vertebrate, mosquito	*Gallus gallus*
Koutango virus	EU082200.2	ABW76844.2	0.549	*Gerbilliscus kempi* (H) [31]<br>*Rhipicephalus* (V) [31]<br>*Hyalomma* (V) [31]<br>*Ornithodoros* (V) [31]<br>*Aedes aegypti* (V) [32]<br>*Homo sapiens* (H) [33]<br>*Mastomys* (H) [33]<br>*Lemniscomys striatus* (H) [33]	MBFV	0.196	0.581	MBFV / Vertebrate, tick	*Bos taurus*
Kunjin virus	JX276662.1	AFR66759.1	0.545	*Culex annulirostis* (V) [34]<br>*Aedes tremulus* (V) [35]<br>*Culex australicus* (V) [36]<br>*Culex squamosus* (V) [37]<br>*Aedes vigilax* (V) [38]<br>*Culex quinquefasciatus* (V) [36]<br>*Homo sapiens* (H) [39]<br>*Equus* (H) [40]<br>Sentinel chicken (H) [41]<br>*Nycticorax caledonicus* (H) [42]<br>*Culex pseudovishnui* (V) [43]<br>*Anatidae* sp. [43]	MBFV	0.197	0.337	MBFV / Vertebrate, mosquito, tick	*Gallus gallus*
Kyasanur forest disease virus	AY323490.1	AAQ91607.1	0.603	*Homo sapiens* (H) [1]<br>*Semnopithecus entellus* (H) [1]<br>*Haemaphysalis spinigera* (V) [44]<br>*Gallus gallus* (H) [45]	TBFV	1.324	0.825	TBFV / Tick, vertebrate	*Ixodes scapularis*
Lammi virus	NC_024806.1	YP_009056848.1	0.518	*Culicidae* (H) [1]	dhIOFV	-0.173	-0.879	dhIOFV / Vertebrate, mosquito	*Columba livia*
Langat virus	NC_003690.1	NP_620108.1	0.592	*Homo sapiens* (H) [1]<br>*Mus* (H) [1]<br>*Ixodes granulatus* (V) [46]<br>*Haemaphysalis Papuana* (V) [47]	TBFV	0.930	1.103	TBFV / Tick, vertebrate	*Sus scrofa*
Louping ill virus	NC_001809.1	NP_044677.1	0.606	*Homo sapiens* (H) [1]<br>*Canis lupus familiaris* (H) [1]<br>*Equus caballus* (H) [1]<br>*Sus scrofa* (H) [1]<br>*Bos taurus* (H) [1]<br>*Ovis aries* (H) [1]<br>*Ixodes ricinus* (V) [1]<br>*Cervinae* (H) [1]	TBFV	1.348	0.758	TBFV / Tick, vertebrate	*Ixodes scapularis*
Meaban virus	NC_033721.1	YP_009345031.1	0.600	*Aves* (H) [1]<br>*Homo sapiens* (H) [1]<br>*Ornithodoros maritimus* (V) [48]	TBFV	1.280	1.020	TBFV / Tick, vertebrate	*Sus scrofa*

Mercadeo virus	NC_027819.1	YP_009164031.1	0.573	*Culex* (H) [1]	IOFV	1.315	-2.140	IOFV / Mosquito	*Aedes albopictus*
Modoc virus	NC_003635.1	NP_619758.1	0.447	*Homo sapiens* (H) [1]<br>*Peromyscus maniculatus* (H) [1]	UVFV	-1.783	-0.187	UVFV / Vertebrate	*Xenopus laevis*
Montana myotis leukoencephalitis virus	NC_004119.1	NP_689391.1	0.415	*Myotis lucifugus* (H) [1]	UVFV	-2.379	-0.290	UVFV / Vertebrate	*Xenopus laevis*
Mosquito flavivirus	NC_021069.1	YP_007877501.1	0.588	*Culex tritaeniorhynchus* (H) [1]	IOFV	1.447	-1.340	IOFV / Mosquito	*Culex quinquefasciatus*
Murray Valley encephalitis virus	NC_000943.1	NP_051124.1	0.493	*Homo sapiens* (H) [1]<br>*Culex annulirostris* (V) [1]	MBFV	-0.637	-0.495	UVFV / Vertebrate, mosquito	*Columba livia*
Naranjal virus	KF917538.1	AIU94742.1	0.516	Sentinel hamster (H) [2]	MBFV	-0.482	0.580	MBFV / Vertebrate	*Homo sapiens*
Negishi virus	KT224355.1	ALP82435.1	0.607	*Homo sapiens* (H) [49]	TBFV	1.388	0.664	TBFV / Tick, vertebrate	*Ixodes scapularis*
New Mapoon virus	NC_032088.1	YP_009328360.1	0.553	*Culicidae* (H) [1]<br>*Culex annulirostris* (H) [1]	MBFV	0.366	0.767	MBFV / Vertebrate, tick	*Bos taurus*
Nounane virus	NC_033715.1	YP_009345019.1	0.531	*Uranotaenia mashonaensis* (H) [2]	dhIOFV	0.048	-1.026	dhIOFV / Vertebrate, mosquito	*Aedes albopictus*
Ntaya virus	NC_018705.3	YP_006846328.2	0.489	*Homo sapiens* (H) [1]<br>*Mus musculus* (H) [1]<br>*Coquillettidia pseudoconopas* (V) [50]<br>*Uranotaenia alboabdominalis* (V) [50]<br>*Culiseta fraseri* (V) [50]<br>*Coquillettidia aurites* (V) [50]<br>*Aedes simpsoni* (V) [50]<br>*Aedes apicoargenteus* (V) [50]<br>*Aedes africanus* (V) [50]<br>*Aedes albomarginatus* (V) [50]<br>*Lutzia tigripes* (V) [50]<br>*Culex poicilipes* (V) [50]<br>*Culex pruina* (V) [50]<br>*Culex moucheti* (V) [50]<br>*Culex* spp. (V) [50]	MBFV	-0.667	-1.116	UVFV / Vertebrate, mosquito	*Anas platyrhynchos*
Ochlerotatus caspius flavivirus	NC_034242.1	YP_009352228.1	0.499	*Ochlerotatus caspius* (H) [1]<br>*Aedes albopictus* (H) [51]	IOFV	-0.136	-1.812	IOFV / Mosquito	*Aedes aegypti*
Omsk hemorrhagic fever virus	NC_005062.1	NP_878909.1	0.577	*Ixodes* (V) [1]<br>*Homo sapiens* (H) [1]<br>*Ondatra zibethicus* (H) [1]<br>*Dermacentor reticulatus* (V) [1]<br>*Arvicola amphibius* (H) [1]	TBFV	0.716	0.523	TBFV / Tick, vertebrate	*Bos taurus*
Palm Creek virus	NC_033694.1	YP_009344962.1	0.527	*Coquillettidia xanthogaster* (H) [1]	IOFV	0.530	-2.403	IOFV / Mosquito	*Aedes aegypti*
Paraiso Escondido virus	NC_027999.1	YP_009169331.1	0.473	*Psathyromyia abonnenci* (H) [1]	MBFV	-0.896	-2.060	UVFV / Mosquito	*Aedes aegypti*
Phnom Penh bat virus	NC_034007.1	YP_009350101.1	0.444	*Cynopterus brachyotis* (H) [1]	UVFV	-1.762	-0.143	UVFV / Vertebrate	*Xenopus laevis*

Potiskum virus	NC_029054.2	YP_009433741.1	0.477	*Culicidae* (V) [1]<br>*Homo sapiens* (H) [1]<br>*Rodentia* (H) [1]<br>*Gallus gallus domesticus* (H) [52]	MBFV	-1.425	0.827	MBFV / Vertebrate	*Alligator mississipiensis*
Powassan virus	NC_003687.1	NP_620099.1	0.574	*Ixodes scapularis* (V) [1]<br>*Homo sapiens* (H) [1]<br>*Marmota monax* (H) [1]<br>*Ixodes spinipalpis* (V) [1]<br>*Dermacentor andersoni* (V) [1]<br>*Ixodes cookei* (V) [1]<br>*Lepus americanus* (H) [1]	TBFV	0.693	0.270	TBFV / Tick, vertebrate, mosquito	*Bos taurus*
Quang Binh virus	NC_012671.1	YP_002884239.1	0.586	*Culicidae* (H) [1]<br>*Culex tritaeniorhynchus* (H) [1]	IOFV	1.608	-2.015	IOFV / Mosquito	*Aedes albopictus*
Rio Bravo virus	NC_003675.1	NP_620044.1	0.407	*Homo sapiens* (H) [1]<br>*Eptesicus fuscus* (H) [1]<br>*Tadarida brasiliensis Mexicana* (H) [1]<br>*Molossus ater* (H) [1]	UVFV	-2.468	-0.908	UVFV / Vertebrate	*Xenopus laevis*
Rocio virus	AY632542.4	AAV34158.1	0.584	*Homo sapiens* (H) [53, 54]<br>*Mus musculus* (H) [53]<br>*Zonotrichia capensis* (H) [53]<br>*Psorophora ferox* (V) [55, 56]<br>*Aedes scapularis* (V) [55]	MBFV	0.779	0.963	MBFV / Vertebrate, tick	*Sus scrofa*
Royal Farm virus	DQ235149.1	ABB90673.1	0.585	*Argas hermanni* (V) [57]<br>*Cricetidae* (H) [57]	TBFV	1.186	-0.021	TBFV / Tick, vertebrate, mosquito	*Anopheles gambiae*
Saboya virus	NC_033697.1	YP_009344967.1	0.477	*Mus musculus* (H) [1]<br>*Jaculus jaculus* (H) [1]<br>*Arvicanthis niloticus* (H) [1]<br>*Mastomys sp.* (H) [1]<br>*Gerbilliscus kempi* (H) [1]<br>*Phlebotomus duboscqi* (V) [58, 59]<br>*Sergentomyia inermis* (V) [59]<br>*Sergentomyia squamipleuris* (V) [59]<br>*Sergentomyia adleri* (V) [59]<br>*Sergentomyia clydei* (V) [59]<br>*Sergentomyia antennata* (V) [59]<br>*Sergentomyia buxtoni* (V) [59]<br>*Sergentomyia dubia* (V) [59]<br>*Sergentomyia schwetzi* (V) [59]<br>*Sergentomyia magna* (V) [59]	MBFV	-1.389	0.636	MBFV / Vertebrate	*Alligator mississipiensis*
Saumarez Reef virus	NC_033726.1	YP_009345037.1	0.576	*Aves* (H) [1]<br>*Homo sapiens* (H) [1]<br>*Ornithodoros capensis* (V) [60]<br>*Ixodes eudyptidis* (V) [60]	TBFV	1.063	-0.027	TBFV / Tick, vertebrate, mosquito	*Anopheles gambiae*
Sepik virus	NC_008719.1	YP_950478.1	0.483	*Culicidae* (V) [1]<br>*Ovis aries* (H) [10]<br>*Homo sapiens* (H) [10]	MBFV	-1.280	0.765	MBFV / Vertebrate	*Alligator mississipiensis*
Siberian tick-borne encephalitis virus	L40361.3	AAF82240.2	0.607	*Ixodes persulcatus* (V) [61]	TBFV	1.414	0.244	TBFV / Tick, vertebrate	*Ixodes scapularis*
Sokoluk virus	NC_026624.1	YP_009126875.1	0.588	*Pipistrellus pipistrellus* (H) [1]	MBFV	0.932	0.597	TBFV / Vertebrate, tick	*Ixodes scapularis*

Spanish goat encephalitis virus	NC_027709.1	YP_009162613.1	0.610	*Capra hircus* (H) [1]	TBFV	1.449	0.635	TBFV / Tick, vertebrate	*Sus scrofa*
Spondweni virus	NC_029055.1	YP_009222008.1	0.580	*Aedes circumluteolus* (V) [1]<br>*Mansonia uniformis* (V) [62]<br>*Homo sapiens* (H) [63, 64]	MBFV	0.794	1.072	MBFV / Vertebrate, tick	*Sus scrofa*
St. Louis encephalitis virus	NC_007580.2	YP_001008348.1	0.524	*Culex quinquefasciatus* (V) [1]<br>*Dromaius novaehollandiae* (H) [1]<br>*Dasypodidae* (H) [1]<br>*Homo sapiens* (H) [1]<br>*Culex nigripalpus* (V) [1]<br>*Passer domesticus* (H) [1]	MBFV	-0.052	-0.494	MBFV / Vertebrate, mosquito	*Columba livia*
Stratford virus	KM225263.1	AIJ19432.1	0.529	*Aedes albopictus* (V) [1]<br>*Macropodidae* (H) [1]<br>*Homo sapiens* (H) [1]<br>*Equus caballus* (H) [1]	MBFV	0.048	-0.146	TBFV / Vertebrate, mosquito	*Gallus gallus*
Tembusu virus	NC_015843.2	YP_004734464.1	0.509	*Anser* sp. (H) [1]<br>*Culex tritaeniorhynchus* (V) [65]<br>*Culex vishnui* (V) [65]<br>*Culex gelidus* (V) [65]<br>*Culex pipiens* (V) [66]	MBFV	-0.518	0.030	UVFV / Vertebrate, mosquito	*Columba livia*
T'Ho virus	NC_034151.1	YP_009351820.1	0.527	*Culex quinquefasciatus* (V) [1]<br>Vertebrates (H) [67]	MBFV	-0.356	0.559	MBFV / Vertebrate	*Homo sapiens*
Torres virus	KM225265.1	AIJ19434.1	0.526	*Culicidae* (V) [1]<br>*Aedes albopictus* (V) [1]<br>*Culex gelidus* (V) [30]<br>*Sus* (H) [30]	MBFV	-0.141	0.306	TBFV / Vertebrate	*Gallus gallus*
Turkish sheep encephalitis virus	DQ235151.1	ABB90675.1	0.613	*Ovis* (H) [68]	TBFV	1.465	0.582	TBFV / Tick, vertebrate	*Ixodes scapularis*
Tyuleniy virus	NC_023424.1	YP_009001464.1	0.579	*Ixodes uriae* (V) [1]	TBFV	1.058	-0.047	TBFV / Tick, vertebrate, mosquito	*Anopheles gambiae*
Uganda S virus	NC_033698.1	YP_009344968.1	0.464	*Mus musculus* (H) [1]<br>*Saxicola rubetra* (H) [1]<br>*Aedes longipalpis* (V) [69]<br>*Aedes ingrami* (V) [69]<br>*Aedes natronius* (V) [69]<br>*Macaca mulatta* (H) [69]	MBFV	-1.442	-0.122	UVFV / Vertebrate	*Anas platyrhynchos*
Usutu virus	NC_006551.1	YP_164264.1	0.551	*Aedes albopictus* (V) [1]<br>*Culex pipiens* (V) [1]<br>*Turdus merula* (H) [1]<br>*Homo sapiens* (H) [1]<br>*Anopheles maculipennis* (V) [1]<br>*Ochlerotatus caspius* (V) [1]<br>*Coquillettidia aurites* (V) [1]<br>*Mansonia Africana* (V) [1]<br>*Culex neavei* (V) [1]<br>*Culex perexiguus* (V) [1]	MBFV	0.325	0.497	MBFV / Vertebrate, tick	*Bos taurus*
Wesselsbron virus	NC_012735.1	YP_002922020.1	0.477	*Aedes* (V) [1]<br>*Homo sapiens* (H) [1]<br>*Capra hircus* (H) [1]<br>*Ovis aries* (H) [1]	MBFV	-1.324	0.237	MBFV / Vertebrate	*Alligator mississipiensis*

West Nile virus lineage 1	NC_009942.1	YP_001527877.1	0.560	*Aedes* (V) [1]<br>*Aves* (H) [1]<br>*Homo sapiens* (H) [1]<br>*Amblyomma variegatum* (V) [1]<br>*Hyalomma marginatum* (V) [1]<br>*Rhipicephalus* (V) [1]<br>*Culex* (V) [1]<br>*Mansonia uniformis* (V) [1]<br>*Mimomyia* (V) [1]<br>*Chlorocebus aethiops* (H) [1]<br>*Mesocricetus auratus* (H) [1]<br>*Bubo scandiacus* (H) [1]<br>*Mus* (H) [1]<br>*Corvidae* [70]	MBFV	0.463	0.248		MBFV / Vertebrate, mosquito, tick	*Bos taurus*
West Nile virus lineage 2	NC_001563.2	NP_041724.2	0.551	*Homo sapiens* (H) [1]	MBFV	0.341	0.112		MBFV / Vertebrate, mosquito, tick	*Gallus gallus*
Western tick-borne encephalitis virus	NC_001672.1	NP_043135.1	0.595	*Homo sapiens* (H) [1]<br>*Mus musculus* (H) [1]<br>*Ixodes ricinus* (V) [1]<br>*Ixodes persulcatus* (V) [1]	TBFV	1.245	0.276		TBFV / Tick, vertebrate	*Ixodes scapularis*
Yaounde virus	NC_034018.1	YP_009350103.1	0.545	*Culex nebulosus*(V) [1]<br>*Culex telesilla* (V) [71]<br>*Culex quiarti* (V) [71]<br>*Eretmapodites oedipodeios* (V) [71]<br>*Aedes aegypti* (V) [71]<br>*Culex perfuscus* (V) [71]<br>*Culex pruina* (V) [71]<br>*Culex duttoni* (V) [71]<br>*Bycanistes sharpie* (H) [71]<br>*Aves* (H) [72]<br>*Praomys* (H) [71]	MBFV	0.338	-0.622		dhIOFV / Vertebrate, mosquito	*Gallus gallus*
Yellow fever virus	NC_002031.1	NP_041726.1	0.537	*Aedes aegypti* (V) [1]<br>*Aedes simpsoni* (V) [1]<br>*Homo sapiens* (H) [1]<br>*Aedes luteocephalus* (V) [1]<br>*Simiiformes* (H) [1]	MBFV	-0.409	1.879		MBFV / Vertebrate	*Mus musculus*
Yokose virus	NC_005039.1	NP_872627.1	0.485	*Miniopterus fuliginosus* (H) [1]<br>*Culicidae* (V) [73]	MBFV	-1.119	0.002		MBFV / Vertebrate	*Alligator mississippiensis*
Zika virus	NC_012532.1	YP_002790881.1	0.541	*Aedes aegypti* (V) [1]<br>*Aedes albopictus* (V) [1]<br>*Macaca mulatta* (H) [1]<br>*Homo sapiens* (H) [1]<br>*Mus musculus* (H) [1]	MBFV	-0.131	1.189		MBFV / Vertebrate	*Myotis brandtii*

[1] Information obtained from Virus-Host Database [74].
[2] Information obtained from GenBank [75].

MBFV = mosquito-borne flavivirus; TBFV = tick-borne flavivirus; IOFV = insect-only flavivirus; UVFV = unknown vector flavivirus; dhIOFV = dual-host IOFV
GC3: Proportion of Guanine+Cytosine at the third position of the codon

Supplementary table S2. Normalized Codon Adaptation Index values of flaviviruses (genus *Flavivirus*) and their putative hosts.

**Virus** **Host**	*Aedes aegypti*	*Aedes albopictus*	*Alligator mississippiensis*	*Anas platyrhynchos*	*Anopheles gambiae*	*Bos taurus*	*Columba livia*	*Culex quinquefasciatus*	*Gallus gallus*	*Homo sapiens*	*Ixodes scapularis*	*Mus musculus*	*Myotis brandtii*	*Myotis davidii*	*Sus scrofa*	*Xenopus laevis*
Aedes flavivirus	0,903	0,866	0,938	0,947	0,687	0,823	0,948	0,729	0,897	0,865	0,706	0,855	0,828	0,794	0,751	0,927
Alfuy virus	0,958	0,923	1,023	1,029	0,739	0,890	1,030	0,772	0,978	0,937	0,746	0,930	0,899	0,861	0,813	1,004
Alkhurma hemorrhagic fever virus	0,976	0,948	1,043	1,047	0,776	0,926	1,051	0,821	1,005	0,968	0,793	0,967	0,934	0,898	0,856	1,011
Anopheles flavivirus variant 1	0,936	0,897	0,973	0,981	0,713	0,849	0,983	0,749	0,931	0,892	0,719	0,884	0,854	0,818	0,774	0,963
Anopheles flavivirus variant 2	0,934	0,894	0,983	0,991	0,710	0,854	0,991	0,746	0,938	0,899	0,720	0,892	0,861	0,825	0,779	0,975
Apoi virus	0,938	0,900	1,026	1,033	0,700	0,887	1,030	0,744	0,970	0,939	0,718	0,932	0,898	0,857	0,809	1,021
Bagaza virus	1,031	0,993	1,109	1,114	0,793	0,966	1,115	0,836	1,059	1,019	0,803	1,008	0,976	0,936	0,885	1,082
Bainyik virus	0,961	0,928	1,035	1,040	0,743	0,901	1,042	0,779	0,987	0,949	0,750	0,942	0,912	0,874	0,826	1,013
Bamaga virus	0,973	0,935	1,069	1,072	0,732	0,917	1,069	0,773	1,011	0,973	0,734	0,964	0,930	0,888	0,837	1,055
Banzi virus	0,977	0,943	1,065	1,067	0,750	0,931	1,070	0,797	1,018	0,981	0,771	0,975	0,942	0,902	0,856	1,037
Bouboui virus	0,949	0,910	1,043	1,047	0,710	0,896	1,043	0,745	0,985	0,950	0,723	0,943	0,907	0,867	0,814	1,036
Bussuquara virus	0,968	0,934	1,041	1,046	0,743	0,907	1,047	0,778	0,994	0,955	0,749	0,946	0,916	0,878	0,829	1,018
Cacipacore virus	0,987	0,949	1,058	1,065	0,753	0,919	1,066	0,791	1,009	0,970	0,766	0,961	0,929	0,888	0,839	1,039
Calbertado virus	1,013	0,977	1,026	1,036	0,786	0,907	1,041	0,836	0,992	0,947	0,799	0,941	0,911	0,877	0,836	0,996
Cell fusing agent virus	0,913	0,879	0,965	0,971	0,710	0,856	0,975	0,753	0,929	0,896	0,736	0,889	0,861	0,830	0,787	0,937
Chaoyang virus	1,012	0,971	1,083	1,091	0,763	0,931	1,091	0,800	1,033	0,984	0,767	0,975	0,940	0,897	0,845	1,067
Culex flavivirus	1,030	0,999	1,050	1,061	0,820	0,939	1,067	0,876	1,024	0,975	0,844	0,971	0,943	0,911	0,871	1,009
Culiseta flavivirus	0,933	0,892	0,978	0,988	0,700	0,845	0,986	0,736	0,929	0,892	0,704	0,881	0,853	0,814	0,767	0,974
Deer tick virus	0,992	0,961	1,065	1,070	0,776	0,938	1,073	0,824	1,022	0,983	0,794	0,978	0,946	0,909	0,865	1,039
Dengue virus 1	0,993	0,949	1,078	1,086	0,737	0,921	1,080	0,764	1,016	0,979	0,743	0,964	0,930	0,886	0,830	1,078
Dengue virus 2	1,014	0,968	1,112	1,122	0,751	0,947	1,115	0,778	1,046	1,008	0,758	0,992	0,959	0,913	0,854	1,111
Dengue virus 3	0,985	0,941	1,092	1,099	0,735	0,932	1,096	0,761	1,031	0,991	0,749	0,976	0,943	0,899	0,841	1,085
Dengue virus 4	0,978	0,937	1,080	1,088	0,734	0,931	1,086	0,765	1,024	0,988	0,750	0,974	0,942	0,899	0,843	1,069
Donggang virus	0,906	0,864	0,979	0,987	0,666	0,838	0,984	0,705	0,927	0,887	0,680	0,881	0,847	0,808	0,760	0,975
Edge Hill virus	0,958	0,920	1,049	1,053	0,718	0,900	1,052	0,758	0,992	0,955	0,721	0,946	0,912	0,869	0,817	1,044
Entebbe bat virus	0,996	0,962	1,068	1,074	0,783	0,945	1,077	0,818	1,027	0,991	0,800	0,979	0,952	0,917	0,870	1,035
Far Eastern tick-borne encephalitis virus	1,000	0,973	1,064	1,069	0,795	0,948	1,074	0,845	1,029	0,988	0,816	0,986	0,955	0,919	0,878	1,029

Fitzroy River Virus	0,964	0,922	1,068	1,074	0,716	0,919	1,072	0,754	1,008	0,976	0,732	0,964	0,931	0,887	0,834	1,060
Gadgets Gully virus	0,996	0,967	1,065	1,072	0,790	0,937	1,076	0,828	1,026	0,983	0,793	0,978	0,945	0,909	0,863	1,035
Hanko virus	0,945	0,906	0,985	0,991	0,706	0,853	0,991	0,750	0,935	0,901	0,702	0,887	0,860	0,821	0,771	0,975
Iguape virus	1,053	1,018	1,122	1,124	0,814	0,989	1,130	0,863	1,077	1,037	0,837	1,027	0,997	0,958	0,908	1,081
Ilheus virus	0,999	0,965	1,074	1,075	0,781	0,949	1,080	0,829	1,035	0,994	0,817	0,990	0,957	0,921	0,877	1,029
Ilomantsi virus	0,923	0,882	0,989	0,998	0,682	0,843	0,995	0,718	0,937	0,894	0,692	0,887	0,852	0,812	0,764	0,989
Israel turkey meningoencephalomyelitis virus	1,015	0,977	1,092	1,097	0,776	0,949	1,099	0,818	1,041	1,000	0,787	0,992	0,959	0,917	0,867	1,069
Japanese encephalitis virus	0,947	0,916	1,004	1,009	0,736	0,886	1,014	0,778	0,965	0,928	0,756	0,922	0,893	0,857	0,815	0,973
Jugra virus	0,959	0,919	1,063	1,067	0,723	0,911	1,063	0,754	1,001	0,968	0,734	0,959	0,923	0,880	0,829	1,057
Jutiapa virus	0,974	0,930	1,073	1,080	0,714	0,914	1,073	0,754	1,005	0,975	0,709	0,960	0,926	0,880	0,823	1,085
Kadam virus	0,966	0,936	1,045	1,048	0,757	0,919	1,051	0,803	1,001	0,964	0,776	0,962	0,927	0,891	0,846	1,018
Kamiti River virus	0,917	0,880	0,960	0,969	0,693	0,845	0,970	0,740	0,918	0,888	0,719	0,883	0,851	0,816	0,775	0,948
Karshi virus	1,005	0,981	1,074	1,074	0,820	0,964	1,082	0,865	1,043	1,004	0,835	1,001	0,970	0,938	0,898	1,025
Kedougou virus	1,012	0,983	1,093	1,093	0,808	0,973	1,099	0,849	1,056	1,016	0,831	1,008	0,980	0,945	0,901	1,042
Kokobera virus	0,980	0,948	1,050	1,056	0,755	0,921	1,059	0,800	1,004	0,968	0,764	0,956	0,930	0,891	0,843	1,027
Koutango virus	1,009	0,974	1,081	1,088	0,774	0,950	1,089	0,822	1,037	0,997	0,805	0,991	0,959	0,920	0,873	1,051
Kunjin virus	0,994	0,960	1,065	1,071	0,770	0,934	1,073	0,813	1,022	0,982	0,786	0,975	0,943	0,905	0,859	1,038
Kyasanur forest disease virus	0,990	0,964	1,050	1,054	0,797	0,941	1,061	0,848	1,019	0,979	0,823	0,979	0,947	0,913	0,872	1,010
Lammi virus	1,012	0,972	1,080	1,087	0,773	0,933	1,087	0,807	1,031	0,984	0,782	0,976	0,941	0,901	0,849	1,057
Langat virus	1,000	0,974	1,074	1,076	0,803	0,956	1,081	0,849	1,039	0,997	0,821	0,996	0,962	0,927	0,885	1,037
Louping ill virus	1,000	0,974	1,062	1,068	0,810	0,949	1,074	0,856	1,032	0,988	0,832	0,987	0,956	0,922	0,883	1,021
Meaban virus	0,956	0,934	1,021	1,024	0,776	0,914	1,030	0,825	0,991	0,952	0,790	0,950	0,920	0,887	0,849	0,979
Mercadeo virus	0,944	0,912	0,964	0,973	0,736	0,855	0,978	0,786	0,932	0,891	0,751	0,884	0,858	0,827	0,788	0,936
Modoc virus	0,988	0,943	1,086	1,091	0,721	0,924	1,086	0,763	1,014	0,986	0,718	0,971	0,937	0,889	0,833	1,096
Montana myotis leukoencephalitis virus	0,974	0,927	1,079	1,086	0,696	0,908	1,077	0,737	1,004	0,974	0,694	0,959	0,923	0,873	0,814	1,099
Mosquito flavivirus	1,001	0,967	1,026	1,036	0,781	0,914	1,040	0,837	0,994	0,953	0,810	0,947	0,919	0,887	0,846	0,990
Murray Valley encephalitis virus	0,972	0,934	1,047	1,055	0,736	0,903	1,054	0,771	0,996	0,955	0,737	0,945	0,913	0,872	0,821	1,036
Naranjal virus	0,974	0,938	1,059	1,063	0,744	0,919	1,064	0,779	1,008	0,972	0,755	0,963	0,929	0,889	0,839	1,039
Negishi virus	0,995	0,970	1,056	1,061	0,807	0,944	1,067	0,854	1,026	0,983	0,827	0,981	0,951	0,917	0,877	1,014

New Mapoon virus	0,921	0,889	0,986	0,991	0,714	0,871	0,995	0,755	0,946	0,913	0,744	0,907	0,877	0,843	0,801	0,960
Nounane virus	0,992	0,953	1,051	1,058	0,760	0,910	1,058	0,792	1,004	0,960	0,770	0,952	0,919	0,881	0,832	1,026
Ntaya virus	1,020	0,980	1,095	1,103	0,770	0,940	1,102	0,806	1,039	0,995	0,770	0,984	0,950	0,907	0,852	1,087
Ochlerotatus caspius flavivirus	0,938	0,899	0,983	0,990	0,704	0,853	0,990	0,749	0,935	0,900	0,708	0,890	0,861	0,823	0,775	0,972
Omsk hemorrhagic fever virus	1,021	0,991	1,091	1,095	0,808	0,964	1,099	0,855	1,050	1,009	0,827	1,007	0,972	0,937	0,893	1,059
Palm Creek virus	0,996	0,957	1,031	1,040	0,767	0,901	1,043	0,810	0,991	0,944	0,769	0,933	0,906	0,868	0,820	1,014
Paraiso Escondido virus	0,996	0,956	1,064	1,073	0,745	0,905	1,069	0,781	1,008	0,960	0,734	0,950	0,915	0,872	0,820	1,065
Phnom Penh bat virus	0,959	0,917	1,054	1,060	0,705	0,899	1,054	0,739	0,987	0,958	0,696	0,942	0,910	0,865	0,809	1,063
Potiskum virus	0,987	0,944	1,090	1,095	0,726	0,934	1,091	0,773	1,026	0,993	0,742	0,983	0,947	0,902	0,847	1,086
Powassan virus	0,996	0,966	1,062	1,066	0,786	0,938	1,070	0,834	1,022	0,982	0,802	0,978	0,946	0,911	0,867	1,032
Quang Binh virus	0,979	0,946	0,992	1,003	0,763	0,885	1,006	0,818	0,961	0,922	0,791	0,918	0,889	0,858	0,818	0,961
Rio Bravo virus	0,988	0,938	1,085	1,094	0,699	0,912	1,085	0,740	1,009	0,979	0,694	0,962	0,926	0,875	0,816	1,110
Rocio virus	1,009	0,978	1,079	1,081	0,793	0,957	1,088	0,841	1,041	1,001	0,825	0,993	0,965	0,929	0,885	1,037
Royal Farm virus	0,987	0,962	1,045	1,050	0,800	0,929	1,056	0,841	1,014	0,969	0,803	0,964	0,936	0,901	0,859	1,009
Saboya virus	0,963	0,921	1,060	1,064	0,709	0,908	1,060	0,753	0,997	0,966	0,722	0,957	0,921	0,878	0,824	1,057
Saumarez Reef virus	0,945	0,920	1,001	1,009	0,751	0,889	1,014	0,806	0,969	0,927	0,773	0,925	0,895	0,861	0,820	0,971
Sepik virus	0,960	0,920	1,056	1,061	0,713	0,908	1,060	0,750	0,997	0,964	0,723	0,951	0,920	0,876	0,825	1,047
Siberian tick-borne encephalitis virus	1,014	0,990	1,068	1,072	0,815	0,955	1,079	0,866	1,035	0,995	0,836	0,992	0,962	0,928	0,887	1,026
Sokoluk virus	0,975	0,945	1,037	1,039	0,780	0,922	1,043	0,813	1,000	0,964	0,790	0,953	0,929	0,895	0,851	0,996
Spanish goat encephalitis virus	1,010	0,987	1,071	1,075	0,821	0,959	1,082	0,868	1,042	0,997	0,841	0,995	0,965	0,932	0,892	1,027
Spondweni virus	0,997	0,966	1,071	1,074	0,792	0,948	1,080	0,834	1,034	0,992	0,815	0,986	0,956	0,920	0,876	1,025
St. Louis encephalitis virus	1,042	1,004	1,113	1,120	0,794	0,966	1,121	0,845	1,063	1,019	0,808	1,011	0,977	0,935	0,884	1,089
Stratford virus	0,994	0,961	1,063	1,068	0,773	0,930	1,071	0,807	1,018	0,978	0,769	0,968	0,939	0,899	0,853	1,037
Tembusu virus	0,981	0,944	1,065	1,069	0,750	0,920	1,069	0,787	1,013	0,973	0,752	0,961	0,930	0,890	0,837	1,047
T'Ho virus	0,991	0,954	1,076	1,080	0,761	0,936	1,080	0,795	1,025	0,987	0,773	0,978	0,945	0,905	0,854	1,055
Torres virus	0,956	0,924	1,031	1,034	0,741	0,900	1,035	0,774	0,982	0,949	0,739	0,940	0,910	0,872	0,825	1,008
Turkish sheep encephalitis virus	1,012	0,988	1,074	1,079	0,823	0,961	1,085	0,871	1,043	0,997	0,845	0,996	0,966	0,933	0,892	1,030
Tyuleniy virus	1,008	0,983	1,068	1,073	0,807	0,948	1,078	0,855	1,031	0,990	0,817	0,986	0,956	0,919	0,875	1,033
Uganda S virus	0,953	0,911	1,045	1,050	0,704	0,890	1,046	0,741	0,982	0,948	0,706	0,936	0,902	0,859	0,805	1,046
Usutu virus	0,965	0,932	1,031	1,037	0,747	0,908	1,040	0,792	0,992	0,953	0,767	0,947	0,917	0,880	0,835	1,000

Wesselsbron virus	0,977	0,934	1,068	1,076	0,719	0,916	1,074	0,761	1,009	0,973	0,733	0,963	0,926	0,884	0,831	1,067
West Nile virus lineage 1	1,006	0,973	1,071	1,076	0,781	0,944	1,079	0,829	1,031	0,990	0,801	0,984	0,952	0,915	0,869	1,038
West Nile virus lineage 2	0,999	0,964	1,063	1,070	0,767	0,934	1,072	0,819	1,022	0,979	0,792	0,974	0,942	0,904	0,859	1,031
Western tick-borne encephalitis virus	0,980	0,954	1,037	1,043	0,789	0,924	1,049	0,835	1,006	0,962	0,805	0,959	0,931	0,898	0,857	0,999
Yaounde virus	1,043	1,007	1,103	1,108	0,803	0,965	1,111	0,849	1,058	1,013	0,817	1,005	0,973	0,934	0,886	1,072
Yellow fever virus	0,955	0,921	1,053	1,055	0,732	0,920	1,054	0,778	1,001	0,970	0,755	0,962	0,930	0,891	0,844	1,026
Yokose virus	0,973	0,930	1,057	1,065	0,725	0,909	1,061	0,756	0,999	0,965	0,732	0,951	0,920	0,878	0,825	1,054
Zika virus	0,999	0,965	1,089	1,092	0,772	0,952	1,094	0,815	1,042	1,001	0,792	0,996	0,962	0,921	0,874	1,060

Supplementary table S3. Codon usage tables of all putative hosts.

	UUU	UCU	UAU	UGU	UUC	UCC	UAC	UGC	UUA	UCA	UAA	UGA	UUG	UCG	UAG	UGG
Aedes aegypti	105803	67316	90691	70139	205867	122844	160830	89653	48482	75347	8409	7820	162093	148951	5239	82534
Aedes albopictus	97145	56914	79108	63514	200599	123298	163078	87769	38157	65567	6720	5873	153673	154171	4553	80333
Alligator mississippiensis	285736	266501	210156	170626	299463	254351	242753	208569	143195	222283	9468	15055	237164	70524	7528	204449
Anas platyrhynchos	153243	135585	95599	88295	149230	126705	126168	110527	73660	112384	1521	2599	115508	37472	1122	100505
Anopheles gambiae	94614	32112	52725	50824	165698	108606	173108	91618	26775	43545	7161	6391	75745	182093	4436	75670
Bos taurus	176217	159245	118803	110703	230161	202850	170291	140579	79353	126638	8272	17113	136301	59386	6476	137993
Columba livia	112853	92944	74363	65581	113012	93945	97512	76376	50686	75022	3715	4794	84780	28616	2286	73187
Culex quinquefasciatus	123786	42919	50760	60923	198511	134088	195233	107214	25108	52078	7306	6787	134792	188984	4659	86930
Gallus gallus	45768	38296	32211	23851	54936	42683	48342	36075	19129	31442	2046	2986	34146	14079	1281	32616
Homo sapiens	189379	171196	132715	117458	216388	196012	161579	135489	86682	139095	5404	9518	143507	50249	4328	134648
Ixodes scapularis	74374	55722	31434	33921	155979	105861	129415	100447	19307	37110	4241	8274	66146	96803	4231	72965
Mus musculus	422153	398250	298518	279729	535439	444041	394074	301384	165150	289799	23403	40148	329668	103815	19126	306619
Myotis brandtii	149256	134443	103108	91596	189819	173931	143371	113452	69465	105333	5078	9823	118397	45721	4583	113559
Myotis davidii	113232	103442	76640	70839	155462	144783	117529	95128	51237	81258	3784	8156	90795	39444	3674	92755
Sus scrofa	18160	14246	12717	10902	27973	21586	22023	16776	6442	10448	883	1739	13518	5607	628	17440
Xenopus laevis	475411	435671	348838	278073	343215	321198	290496	239628	259966	343805	19075	17534	332659	77483	9401	245817

	CUU	CCU	CAU	CGU	CUC	CCC	CAC	CGC	CUA	CCA	CAA	CGA	CUG	CCG	CAG	CGG
Aedes aegypti	81073	70190	92779	70386	92845	78473	111503	74523	69643	116049	154187	94417	248006	143421	198592	92034
Aedes albopictus	73743	63435	85963	66043	91059	79507	115067	74949	67101	105136	142086	89873	262230	158240	205119	103979
Alligator mississippiensis	234983	286349	199397	80956	261744	253141	224817	142817	134363	313100	247331	88040	592915	94622	565858	148393
Anas platyrhynchos	122813	143338	94079	42336	135241	121613	119654	60556	60117	145719	122456	45366	278261	46915	271533	61272
Anopheles gambiae	51540	33672	71298	60653	110524	79021	127400	144786	51475	75633	91709	56430	341455	213374	255693	126863
Bos taurus	137259	183974	106989	47991	226797	238009	174809	122283	69555	174268	124199	67416	452888	98259	381244	137128
Columba livia	83984	95097	66456	29591	101401	92973	87675	45990	38718	99227	82943	32668	206622	37795	189692	48921
Culex quinquefasciatus	64004	42642	58083	58474	126302	97230	150387	115735	44253	87713	119463	75642	342737	212800	255462	143812
Gallus gallus	33708	41672	25885	14682	45753	46097	39081	28305	16211	42767	33018	14339	104699	21091	88743	26453
Homo sapiens	147569	198345	123609	49921	212802	225420	168062	115976	79488	192119	140427	68859	437308	81354	387120	129331
Ixodes scapularis	66042	55208	36505	40240	159231	120308	116979	101295	31749	59161	59065	44503	233226	106684	169406	86673
Mus musculus	329757	450637	260637	114854	495018	446868	375626	229758	198032	423707	293318	161412	969515	151521	836320	250836
Myotis brandtii	114062	157094	94505	38894	185980	195453	149357	88661	60061	148475	111991	56543	375305	64984	332354	107579
Myotis davidii	86828	124280	72079	30930	154989	167094	125963	77771	45385	116015	84072	44250	317889	60214	271506	94037
Sus scrofa	13109	18561	9900	4792	27053	25796	18267	14145	6653	16692	11567	6519	53901	9860	40912	13943
Xenopus laevis	378042	377145	298817	119961	274223	244929	267467	120706	213125	432029	395345	122409	540897	91856	585594	119667

	AUU	ACU	AAU	AGU	AUC	ACC	AAC	AGC	AUA	ACA	AAA	AGA	AUG	ACG	AAG	AGG
Aedes aegypti	152166	86616	166027	105612	207964	148111	221033	123334	76072	84025	220087	53146	189682	133677	268281	43987
Aedes albopictus	137495	76150	147062	100974	208043	153503	225036	125677	67644	75402	206848	47629	186399	137826	273900	44683
Alligator mississippiensis	289540	245848	311827	221332	318302	263582	322932	326064	160920	294026	477989	230179	377994	97321	532404	200385
Anas platyrhynchos	141066	117055	148135	106071	153436	121061	168842	167445	80265	148079	247562	118152	173849	57102	251100	107972
Anopheles gambiae	92725	39481	100091	64028	202044	140594	219692	156991	61791	59333	126145	23006	166014	201293	262509	21939
Bos taurus	159826	133607	163964	129267	234653	210799	212732	224422	76928	150192	251908	130360	233655	79271	350736	134871
Columba livia	104393	80840	107072	73499	121220	92966	128058	113525	58143	104091	182096	84864	131436	42191	189730	74351
Culex quinquefasciatus	128311	53265	99723	87381	246469	173860	278300	147234	39912	52962	149090	36161	187954	191512	336380	40696
Gallus gallus	45653	36078	46039	30390	59906	44951	61099	54867	23805	43884	74256	33289	62972	20943	93393	31945
Homo sapiens	175259	147134	190114	139465	220634	203602	206688	220505	82922	167136	278169	133268	236510	66200	353825	131616
Ixodes scapularis	57705	45993	45690	42553	134295	111797	147940	121031	31509	54105	78016	39079	127674	107148	208833	78311
Mus musculus	377698	335039	382284	311331	552184	465115	499149	483013	180467	391437	537723	297135	559953	138180	825270	299472
Myotis brandtii	139814	114452	148430	113992	199193	180990	185755	185631	66531	131463	226768	109231	204831	65277	313098	118086
Myotis davidii	105319	86591	110660	87677	163717	149818	150586	156457	49298	100435	169437	82710	162715	57159	249402	96344
Sus scrofa	15660	13038	16598	11082	28754	26456	25606	23327	7191	14403	23681	12052	25610	9022	38617	13258
Xenopus laevis	470669	372157	520334	336460	352434	296177	419539	337006	300000	463817	720130	342826	502530	95128	601326	252104

	GUU	GCU	GAU	GGU	GUC	GCC	GAC	GGC	GUA	GCA	GAA	GGA	GUG	GCG	GAG	GGG
Aedes aegypti	128092	123462	238605	116851	115996	177203	192964	115501	75096	112842	295762	166203	173024	103277	220013	58749
Aedes albopictus	120907	114500	230285	116327	117235	184026	202864	117142	71956	106383	283652	156762	180844	114481	230877	62953
Alligator mississippiensis	220062	336797	425108	182259	214803	357261	392225	290968	146333	328225	560811	297308	410333	94451	643968	250922
Anas platyrhynchos	117470	181596	201191	94731	105498	153253	180425	133781	74738	173629	275108	151883	198074	45370	284768	115330
Anopheles gambiae	64418	75543	173980	125515	109118	190816	212737	204626	60279	106001	179852	87574	229677	195713	283970	78940
Bos taurus	113850	193365	220484	111861	168797	326809	288879	256275	70781	160241	302609	174863	312161	100065	441648	185928
Columba livia	84579	119203	148911	69163	84576	113404	141019	95801	50144	110554	201781	109153	151071	39420	213638	86177
Culex quinquefasciatus	113170	86702	170362	108252	153568	225445	270497	167341	47010	81107	221992	143559	218927	184999	313182	89461
Gallus gallus	35593	56528	68683	30898	36917	62202	67783	53631	21277	51713	84178	47765	76624	24768	111123	43513
Homo sapiens	121302	204091	246943	117456	155761	311996	278549	247607	78882	178106	336665	183190	305878	84501	451726	182999
Ixodes scapularis	56833	73718	66403	60167	135465	195993	232841	161342	31338	81570	121651	88146	190498	115433	225468	82020
Mus musculus	262535	491093	515049	280522	377902	637878	638504	520069	182733	388723	661498	411344	696158	157124	965963	372099
Myotis brandtii	97606	165822	196915	92699	140631	262234	251134	199899	60268	140268	275392	144558	268064	65575	386908	155884
Myotis davidii	74166	130534	149620	72558	116940	225972	209517	171189	45056	110134	205844	112003	222467	60004	317761	132602
Sus scrofa	10755	19642	22309	11651	20245	36983	33278	29987	6447	15141	27310	18686	38572	10339	48046	21555
Xenopus laevis	351271	411579	613288	253098	244083	319491	432347	265240	240447	424584	772321	441213	434149	85444	662177	270557

Supplementary table S4. Sensitivity of nCAI predictions and observations from supplementary table 1

Virus Group		Observed +	Observed -
Predicted	+	75	19
	-	19	357

dhIOFV		+	-
Predicted	+	5	5
	-	0	84

IOFV		+	-
Predicted	+	11	0
	-	3	80

MBFV		+	-
Predicted	+	34	2
	-	15	43

TBFV		+	-
Predicted	+	20	4
	-	0	70

UVFV		+	-
Predicted	+	5	8
	-	1	80

Host type		Observed +	Observed -
Predicted	+	112	46
	-	1	180

Mosquito		+	-
Predicted	+	19	23
	-	1	70

Tick		+	-
Predicted	+	19	15
	-	0	79

Vertebrate		+	-
Predicted	+	74	8
	-	0	31

Group		Observed +	Observed -
Predicted	+	TP	FP
	-	FN	TN

** TP: True Positives; FP: False Positives; FN: False Negatives; TN: True Negatives.*

References

1. Hoshino K, Isawa H, Tsuda Y, Sawabe K, Kobayashi M. Isolation and characterization of a new insect flavivirus from Aedes albopictus and Aedes flavopictus mosquitoes in Japan. Virology. 2009;391:119–129. doi:10.1016/j.virol.2009.06.025.

2. Doherty RL, Standfast HA, Domrow R, Wetters EJ, Whitehead RH, Carley JG. Studies of the epidemiology of arthropod-borne virus infections at Mitchell River Mission, Cape York Peninsula, North Queensland IV. Arbovirus infections of mosquitoes and mammals, 1967–1969. Trans R Soc Trop Med Hyg. 1971;65:504–513. doi:10.1016/0035-9203(71)90161-1.

3. Doherty RL, Carley JG, Kay BH, Filippich C, Marks EN, Frazier CL. Isolation of virus strains from mosquitoes collected in Queensland, 1972-1976. Aust J Exp Biol Med Sci. 1979;57:509–520.

4. Johansen CA, Nisbet DJ, Zborowski P, van den Hurk AF, Ritchie SA, Mackenzie JS. Flavivirus isolations from mosquitoes collected from western Cape York Peninsula, Australia, 1999-2000. J Am Mosq Control Assoc. 2003;19:392–396.

5. Charrel RN, Zaki AM, Fakeeh M, Yousef AI, de Chesse R, Attoui H, et al. Low diversity of Alkhurma hemorrhagic fever virus, Saudi Arabia, 1994-1999. Emerging Infect Dis. 2005;11:683–688. doi:10.3201/eid1105.041298.

6. Zaki AM. Isolation of a flavivirus related to the tick-borne encephalitis complex from human cases in Saudi Arabia. Trans R Soc Trop Med Hyg. 1997;91:179–181.

7. Bondre VP, Sapkal GN, Yergolkar PN, Fulmali PV, Sankararaman V, Ayachit VM, et al. Genetic characterization of Bagaza virus (BAGV) isolated in India and evidence of anti-BAGV antibodies in sera collected from encephalitis patients. J Gen Virol. 2009;90 Pt 11:2644–2649. doi:10.1099/vir.0.012336-0.

8. Agüero M, Fernández-Pinero J, Buitrago D, Sánchez A, Elizalde M, San Miguel E, et al. Bagaza virus in partridges and pheasants, Spain, 2010. Emerging Infect Dis. 2011;17:1498–1501. doi:10.3201/eid1708.110077.

9. Colmant AMG, Bielefeldt-Ohmann H, Hobson-Peters J, Suen WW, O’Brien CA, van den Hurk AF, et al. A newly discovered flavivirus in the yellow fever virus group displays restricted replication in vertebrates. J Gen Virol. 2016;97:1087–1093. doi:10.1099/jgv.0.000430.

10. Grard G, Moureau G, Charrel RN, Holmes EC, Gould EA, de Lamballerie X. Genomics and evolution of Aedes-borne flaviviruses. J Gen Virol. 2010;91 Pt 1:87–94. doi:10.1099/vir.0.014506-0.

11. Shope RE. The use of a microhemagglutination-inhibition test to follow antibody response after arthropod-borne virus infection in a community of forest animals. An Microbiol. 1963;11:167–169.

12. Gomes G, Causey OR. Bussuquara, A New Arthropod-Borne Virus. Exp Biol Med. 1959;101:275–279. doi:10.3181/00379727-101-24909.

13. Batista WC, Tavares G da SB, Vieira DS, Honda ER, Pereira SS, Tada MS. Notification of the first isolation of Cacipacore virus in a human in the State of Rondônia, Brazil. Rev Soc Bras Med Trop. 2011;44:528–530. doi:10.1590/S0037-86822011000400028.

14. Bolling BG, Eisen L, Moore CG, Blair CD. Insect-specific flaviviruses from Culex mosquitoes in Colorado, with evidence of vertical transmission. Am J Trop Med Hyg. 2011;85:169–177. doi:10.4269/ajtmh.2011.10-0474.

15. Cook S, Bennett SN, Holmes EC, De Chesse R, Moureau G, de Lamballerie X. Isolation of a new strain of the flavivirus cell fusing agent virus in a natural mosquito population from Puerto Rico. J Gen Virol. 2006;87 Pt 4:735–748. doi:10.1099/vir.0.81475-0.

16. Hayasaka D, Ivanov L, Leonova GN, Goto A, Yoshii K, Mizutani T, et al. Distribution and characterization of tick-borne encephalitis viruses from Siberia and far-eastern Asia. J Gen Virol. 2001;82 Pt 6:1319–1328. doi:10.1099/0022-1317-82-6-1319.

17. Johansen CA, Williams SH, Melville LF, Nicholson J, Hall RA, Bielefeldt-Ohmann H, et al. Characterization of fitzroy river virus and serologic evidence of human and animal infection. Emerging Infect Dis. 2017;23:1289–1299. doi:10.3201/eid2308.161440.

18. St George TD, Doherty RL, Carley JG, Filippich C, Brescia A, Casals J, et al. The isolation of arboviruses including a new flavivirus and a new Bunyavirus from Ixodes (Ceratixodes) uriae (Ixodoidea: Ixodidae) collected at Macquarie Island, Australia, 1975-1979. Am J Trop Med Hyg. 1985;34:406–412. doi:10.4269/ajtmh.1985.34.406.

19. Huhtamo E, Moureau G, Cook S, Julkunen O, Putkuri N, Kurkela S, et al. Novel insect-specific flavivirus isolated from northern Europe. Virology. 2012;433:471–478. doi:10.1016/j.virol.2012.08.038.

20. Coimbra TL, Nassar ES, Nagamori AH, Ferreira IB, Pereira LE, Rocco IM, et al. Iguape: a newly recognized flavivirus from São Paulo State, Brazil. Intervirology. 1993;36:144–152. doi:10.1159/000150333.

21. Braverman Y, Davidson I, Chizov-Ginzburg A, Chastel C. Detection of Israel Turkey Meningo-encephalitis Virus from Mosquito (Diptera: Culicidae) and*Culicoides* (Diptera: Ceratopogonidae) Species and Its Survival in*Culex pipiens* and*Phlebotomus papatasi* (Diptera: Phlebotomidae). J Med Entomol. 2003;40:518–521. doi:10.1603/0022-2585-40.4.518.

22. Braverman Y, Boreham PFL, Galun R, Ziv M. The origin of blood meals of biting midges (Diptera: Ceratopogonidae) and mosquitoes (Diptera: Culicidae) trapped in turkey runs in Israel. Rhod J Agric Res. 1977.

23. Henderson BE, Tukei PM, Mccrae AWR, Ssenkubuge Y, Mugo WN. Virus isolations from ixodid ticks in Uganda. Part II. Kadam virus-a new member of arbovirus group B isolated from Rhipicephalus pravus Donitz. East African Medical Journal. 1970.

24. Davies FG. Nairobi sheep disease in Kenya. The isolation of virus from sheep and goats, ticks and possible maintenance hosts. J Hyg. 1978;81:259. doi:10.1017/S0022172400025092.

25. Wood OL, Moussa MI, Hoogstraal H, Büttiker W. Kadam Virus (Togaviridae, Flavivirus) Infecting Camel-Parasitizing Hyalomma Dromedarii Ticks (Acari: Ixodidae) in Saudi Arabia12. J Med Entomol. 1982;19:207–208. doi:10.1093/jmedent/19.2.207.

26. Mugo WN, Shope RE. Kadam virus: Neutralization studies and laboratory transmission by dermacentor variabilis. Trans R Soc Trop Med Hyg. 1972;66:300–304. doi:10.1016/0035-9203(72)90162-9.

27. Lvov DK, Neronov VM, Gromashevsky VL, Skvortsova TM, Berezina LK, Sidorova GA, et al. Karshi" virus, a new flavivirus (Togaviridae) isolated fromOrnithodoros papillipes (Birula, 1895) ticks in Uzbek S.S.R. Arch Virol. 1976;50:29–36. doi:10.1007/BF01317998.

28. Fontenille D, Traore-Lamizana M, Diallo M, Thonnon J, Digoutte JP, Zeller HG. New vectors of Rift Valley fever in West Africa. Emerging Infect Dis. 1998;4:289–293. doi:10.3201/eid0402.980218.

29. Robin Y, Cornet M, Le Gonidec G, Chateau R, Heme G. Kedougou virus (Ar D14701): a new arbovirus (flavivirus) isolated in Senegal. Ann Microbiol (Inst Pasteur). 1978.

30. Johansen CA, Nisbet DJ, Foley PN, Van Den Hurk AF, Hall RA, Mackenzie JS, et al. Flavivirus isolations from mosquitoes collected from Saibai Island in the Torres Strait, Australia, during an incursion of Japanese encephalitis virus. Med Vet Entomol. 2004;18:281–287. doi:10.1111/j.0269-283X.2004.00510.x.

31. Fields BN, Knipe DM, Howley PM, Griffin DE. Chapter 33 Flaviviruses. In: Fields Virology. 4th edition. Philadelphia: Lippincott Williams & Wilkins; 2001. p. 891.

32. Coz J, Valade M, Cornet M, Robin Y. [Transovarian transmission of a Flavivirus, the Koutango virus, in Aedes aegypti L]. C R Acad Sci Hebd Seances Acad Sci D. 1976;283:109–110.

33. Traoré-Lamizana M, Fontenille D, Diallo M, Bâ Y, Zeller HG, Mondo M, et al. Arbovirus surveillance from 1990 to 1995 in the Barkedji area (Ferlo) of Senegal, a possible natural focus of Rift Valley fever virus. J Med Entomol. 2001;38:480–492. doi:10.1603/0022-2585-38.4.480.

34. Marshall ID, Woodroofe GM, Hirsch S. Viruses recovered from mosquitoes and wildlife serum collected in the murray valley of south-eastern australia, february 1974, during an epidemic of encephalitis. Aust J Exp Biol Med. 1982;60:457–470. doi:10.1038/icb.1982.51.

35. Liehne CG, Leivers S, Stanley NF, Alpers MP, Paul S, Liehne PF, et al. Ord River arboviruses--isolations from mosquitoes. Aust J Exp Biol Med Sci. 1976;54:499–504.

36. Russell RC. Mosquito-borne arboviruses in Australia: the current scene and implications of climate change for human health. Int J Parasitol. 1998;28:955–969. doi:10.1016/S0020-7519(98)00053-8.

37. Doherty RL, Whitehead RH, Judith Wetters E, Gorman BM. Studies of the epidemiology of arthropod-borne virus infections at Mitchell River Mission, Cape York Peninsula, North Queensland. Trans R Soc Trop Med Hyg. 1968;62:430–438. doi:10.1016/0035-9203(68)90095-3.

38. Kay BH, Carley JG, Filippich C. The multiplication of queensland and new guinean arboviruses in culex annulirostris skuse and aedes vigilax (skuse) (diptera: culicidae). J Med Entomol. 1975;12:279–283. doi:10.1093/jmedent/12.3.279.

39. Mackenzie JS, Smith DW, Broom AK, Bucens MR. Australian encephalitis in Western Australia, 1978-1991. Med J Aust. 1993;158:591–595.

40. Frost MJ, Zhang J, Edmonds JH, Prow NA, Gu X, Davis R, et al. Characterization of virulent West Nile virus Kunjin strain, Australia, 2011. Emerging Infect Dis. 2012;18:792–800. doi:10.3201/eid1805.111720.

41. Department of Health | National Arbovirus and Malaria Advisory Committee (NAMAC) annual reports. http://www.health.gov.au/internet/main/publishing.nsf/content/cda-arboanrep.htm. Accessed 20 Jul 2018.

42. Boyle DB, Dickerman RW, Marshall ID. Primary viraemia responses of herons to experimental infection with murray valley encephalitis, kunjin and japanese encephalitis viruses. Aust J Exp Biol Med. 1983;61:655–664. doi:10.1038/icb.1983.62.

43. Bowen ETW, Simpson DIH, Platt GS, Way HJ, Smith CEG, Ching CY, et al. Arbovirus infections in Sarawak: The isolation of Kunjin virus from mosquitoes of the*Culex pseudovishnui* group. Annals of Tropical Medicine & Parasitology. 1970;64:263–268. doi:10.1080/00034983.1970.11686690.

44. Trapido H, Rajagopalan PK, Work TH, Varma MG. Kyasanur Forest disease. VIII. Isolation of Kyasanur Forest disease virus from naturally infected ticks of the genus Haemaphysalis. Indian J Med Res. 1959;47:133–138.

45. Varma MG, Webb HE, Pavri KM. Studies on the transmission of Kyasanur Forest disease virus by Haemaphysalis spinigera Newman. Trans R Soc Trop Med Hyg. 1960;54:509–516. doi:10.1016/0035-9203(60)90024-9.

46. Gordon Smith CE. A Virus Resembling Russian Spring–Summer Encephalitis Virus from an Ixodid Tick in Malaya. Nature. 1956;178:581–582. doi:10.1038/178581a0.

47. Bancroft WH, Scott RM, Snitbhan R, Weaver RE, Gould DJ. Isolation of Langat virus from *Haemaphysalis papuana* Thorell in Thailand. Am J Trop Med Hyg. 1976;25:500–504. doi:10.4269/ajtmh.1976.25.500.

48. Arnal A, Gómez-Díaz E, Cerdà-Cuéllar M, Lecollinet S, Pearce-Duvet J, Busquets N, et al. Circulation of a Meaban-like virus in yellow-legged gulls and seabird ticks in the western Mediterranean basin. PLoS One. 2014;9:e89601. doi:10.1371/journal.pone.0089601.

49. Ando K, Kuratsuka K, Arima S, Hironaka N, Honda Y, Ishii K. Studies on the Viruses isolated during Epidemic of Japanese B Encephalitis in 948 in Tokyo Area. Kitasato Archives of Experimental Medicine. 1952.

50. Smithburn KC, Haddow AJ. Ntaya virus; a hitherto unknown agent isolated from mosquitoes collected in Uganda. Proc Soc Exp Biol Med. 1951;77:130–133.

51. Ferreira DD, Cook S, Lopes Â, de Matos AP, Esteves A, Abecasis A, et al. Characterization of an insect-specific flavivirus (OCFVPT) co-isolated from Ochlerotatus caspius collected in southern Portugal along with a putative new Negev-like virus. Virus Genes. 2013;47:532–545. doi:10.1007/s11262-013-0960-9.

52. Omilabu SA, Fagbami AH, Olaleye OD. Susceptibility of laboratory and domestic animals to experimental infection with Potiskum virus. Microbios. 1989;60:53–58.

53. de Souza Lopes O, Coimbra TL, de Abreu Sacchetta L, Calisher CH. Emergence of a new arbovirus disease in Brazil. I. Isolation and characterization of the etiologic agent, Rocio virus. Am J Epidemiol. 1978;107:444–449.

54. de Souza Lopes O, de Abreu Sacchetta L, Coimbra TL, Pinto GH, Glasser CM. Emergence of a new arbovirus disease in Brazil. II. Epidemiologic studies on 1975 epidemic. Am J Epidemiol. 1978;108:394–401.

55. Mitchell CJ, Forattini OP, Miller BR. Vector competence experiments with Rocio virus and three mosquito species from the epidemic zone in Brazil. Rev Saúde Pública. 1986;20:171–177. doi:10.1590/S0034-89101986000300001.

56. de Souza Lopes O, de Abreu Sacchetta L, Francy DB, Jakob WL, Calisher CH. Emergence of a new arbovirus disease in Brazil. III. Isolation of Rocio virus from Psorophora Ferox (Humboldt, 1819). Am J Epidemiol. 1981;113:122–125.

57. Williams RE, Casals J, Moussa MI, Hoogstraal H. Royal farm virus: a new tickborne group B agent related to the RSSE complex. Am J Trop Med Hyg. 1972;21:582–586. doi:10.4269/ajtmh.1972.21.582.

58. Ba Y, Trouillet J, Thonnon J, Fontenille D. Phlebotomus of Senegal: survey of the fauna in the region of Kedougou. Isolation of arbovirus. Bull Soc Pathol Exot. 1999;92:131–135.

59. Fontenille D, Traore-Lamizana M, Trouillet J, Leclerc A, Mondo M, Ba Y, et al. First isolations of arboviruses from phlebotomine sand flies in West Africa. Am J Trop Med Hyg. 1994;50:570–574. doi:10.4269/ajtmh.1994.50.570.

60. St George TD, Standfast HA, Doherty RL, Carley JG, Fillipich C, Brandsma J. The isolation of Saumarez Reef virus, a new flavivirus, from bird ticks Ornithodoros capensis and Ixodes eudyptidis in Australia. Aust J Exp Biol Med Sci. 1977;55:493–499.

61. Liu R, Zhang G, Liu X, Li Y, Zheng Z, Sun X, et al. Detection of the Siberian Tick-borne Encephalitis Virus in the Xinjiang Uygur Autonomous Region, northwestern China. Bing Du Xue Bao. 2016;32:26–31.

62. Kokernot RH, Smithburn KC, Muspratt J, Hodgson B. Studies on arthropod-borne viruses of Tongaland. VIII. Spondweni virus, an agent previously unknown, isolated from Taeniorhynchus (Mansonioides) uniformis Theo. South African Journal of Medical Sciences. 1957.

63. Wolfe MS, Calisher CH, McGuire K. Spondweni virus infection in a foreign resident of Upper Volta. Lancet. 1982;2:1306–1308. doi:10.1016/S0140-6736(82)91511-2.

64. Haddow AJ, Williams MC, Woodall JP, Simpson DI, Goma LK. Twelve isolations of zika virus from aedes (stegomyia) africanus (theobald) taken in and above a uganda forest. Bull World Health Organ. 1964;31:57–69.

65. Leake CJ, Ussery MA, Nisalak A, Hoke CH, Andre RG, Burke DS. Virus isolations from mosquitoes collected during the 1982 Japanese encephalitis epidemic in northern Thailand. Trans R Soc Trop Med Hyg. 1986;80:831–837. doi:10.1016/0035-9203(86)90397-4.

66. Tang Y, Diao Y, Chen H, Ou Q, Liu X, Gao X, et al. Isolation and genetic characterization of a tembusu virus strain isolated from mosquitoes in Shandong, China. Transbound Emerg Dis. 2015;62:209–216. doi:10.1111/tbed.12111.

67. Farfan-Ale JA, Loroño-Pino MA, Garcia-Rejon JE, Hovav E, Powers AM, Lin M, et al. Detection of RNA from a novel West Nile-like virus and high prevalence of an insect-specific flavivirus in mosquitoes in the Yucatan Peninsula of Mexico. Am J Trop Med Hyg. 2009;80:85–95.

68. Hartley WJ, Martin WB, Hakiolu F, Chifney ST. A viral encephalitis of sheep in Turkey. Pendik Institute Journal. 1969;2:89–100.

69. Dick GWA, Haddow AJ. Uganda S virus. A hitherto unrecorded virus isolated from mosquitoes in Ugnada. (I). Isolation and pathogenicity. Trans R Soc Trop Med Hyg. 1952;46:600–618. doi:10.1016/0035-9203(52)90021-7.

70. Lanciotti RS, Roehrig JT, Deubel V, Smith J, Parker M, Steele K, et al. Origin of the West Nile virus responsible for an outbreak of encephalitis in the northeastern United States. Science. 1999;286:2333–2337. doi:10.1126/science.286.5448.2333.

71. Arbovirus Catalog: Yaounde (YAOV). https://wwwn.cdc.gov/arbocat/VirusDetails.aspx?ID=528&SID=7. Accessed 23 Jul 2018.

72. Williams, Richard A. J. Yaoundé-like virus in resident wild bird, Ghana. Afr J Microbiol Res. 2012;6. doi:10.5897/AJMR11.479.

73. Tajima S, Takasaki T, Matsuno S, Nakayama M, Kurane I. Genetic characterization of Yokose virus, a flavivirus isolated from the bat in Japan. Virology. 2005;332:38–44. doi:10.1016/j.virol.2004.06.052.

74. Virus-Host DB. Virus-Host Database. 2017. http://www.genome.jp/virushostdb/. Accessed 6 Oct 2017.

75. Benson DA, Cavanaugh M, Clark K, Karsch-Mizrachi I, Lipman DJ, Ostell J, et al. GenBank. Nucleic Acids Res. 2013;41 Database issue:D36–42. doi:10.1093/nar/gks1195.
