# Supplementary material for: An Unsupervised Algorithm for Host Identification in Flaviviruses
Source: Life (Basel). 2021 May 14;11(5):442. doi: 10.3390/life11050442 (PMC8157105; doi:10.3390/life11050442)
Supplement: Supplementary file 1 [file life-11-00442-s001.zip › supplementary/Truong_MDPI_Supplementary_Figures_rev.pdf]

SUPPLEMENTARY FIGURES

An unsupervised algorithm for host identification in flaviviruses

Phuoc Truong Nguyen [1, 2], Santiago Garcia-Vallve [3], Pere Puigbò [1, 4, 5, *]

[1] *Department of Biology, University of Turku, Turku, Finland.*
[2] *Currently at the Department of Virology, Medicum, Faculty of Medicine, University of Helsinki, Helsinki, Finland.*
[3] *Research Group in Cheminformatics & Nutrition, Department of Biochemistry and Biotechnology, Rovira i Virgili University, Tarragona, Catalonia, Spain*
[4] *Currently at the Department of Biochemistry and Biotechnology, Rovira i Virgili University, Tarragona, Catalonia, Spain*
[5] *Currently at the Nutrition and Health Unit, Eurecat Technology Centre of Catalonia, Reus, Catalonia, Spain*

[*] *Corresponding author: pepuav@utu.fi*

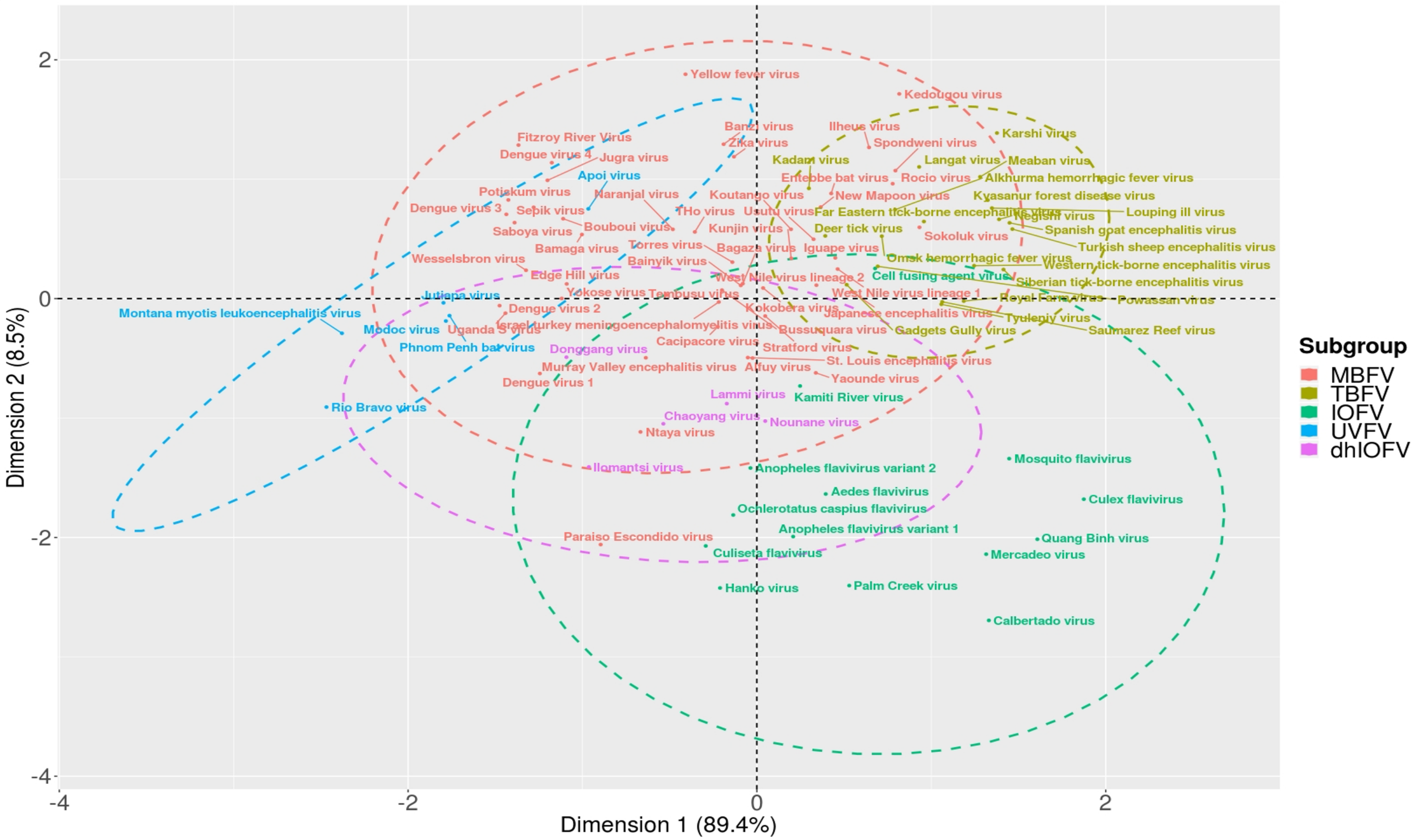

Supplementary figure S1. Interspecies correspondence analysis and subgroup centroids of normalized Codon Adaptation Index (nCAI) values of flaviviruses, genus *Flavivirus* (N = 94). While there is a distinct separation between the clusters of insect-only and the other flavivirus subgroups, the clouds (shown as dashed circles with colors corresponding to a flavivirus subgroup) computed from them show major overlap, indicating that most of the subgroups share similar host preferences, e.g. unknown vector flaviviruses most likely have similar hosts as mosquito-borne flaviviruses. An interesting case can be observed with Paraiso Escondido virus, which is located outside the mosquito-borne cloud, which suggests that it does not have a vertebrate host. This is supported by previous studies, in which the virus is described to infect only sandflies. MBFV = Mosquito-borne flavivirus, TBFV = Tick-borne flavivirus, IOFV = Insect-only flavivirus, UVFV = Unknown vector flavivirus, and dhIOFV = Dual-host insect-only flavivirus. The centroids were computed based on the multivariate normal distribution of each subgroup with a confidence level of 0.95. Dimension 1 explains 89.4 percent and dimension 2 contributes to 8.5 percent of the variation.

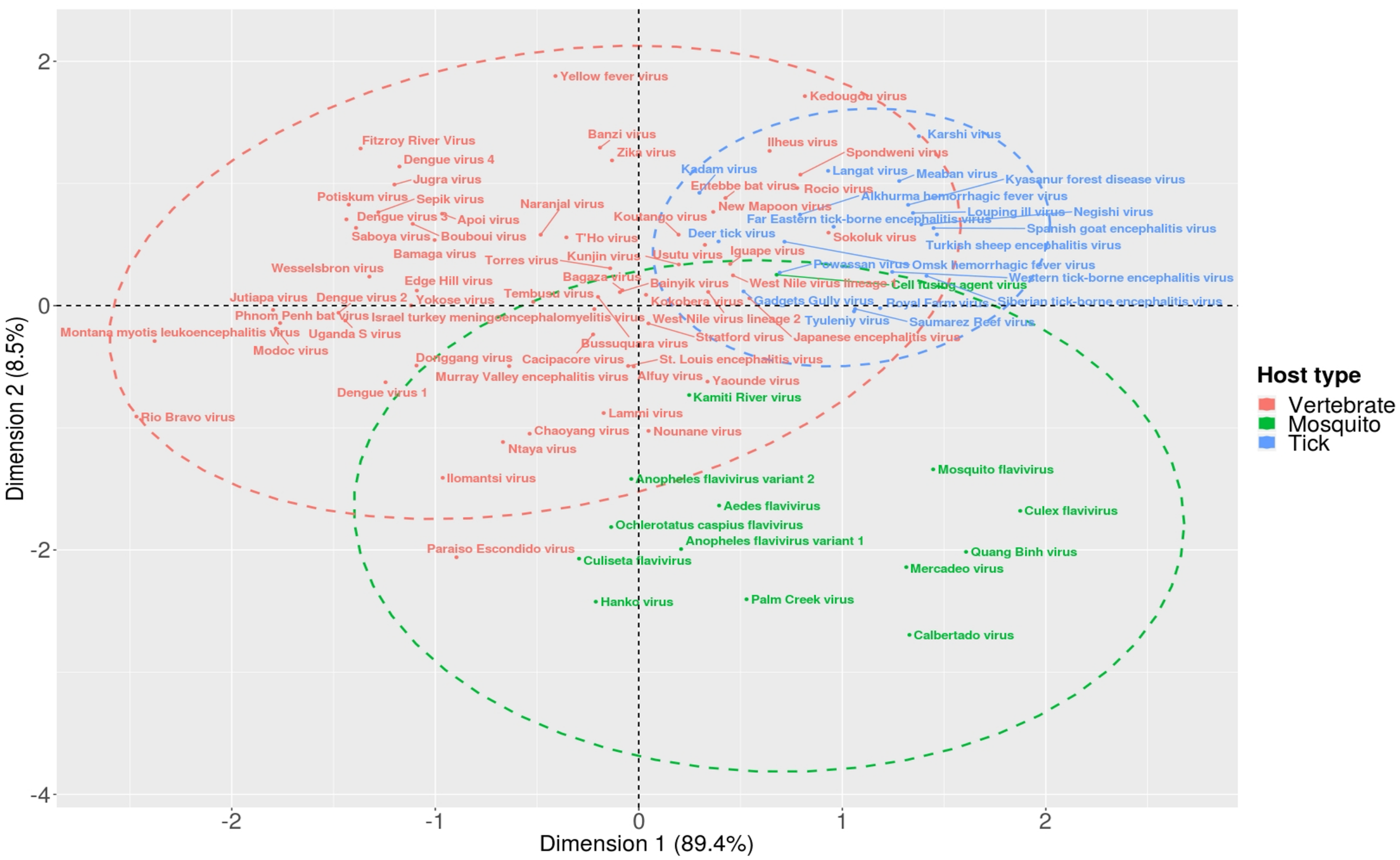

Supplementary figure S2. Interspecies correspondence analysis with host type centroids of normalized Codon Adaptation Index (nCAI) values of flaviviruses, genus *Flavivirus* (N = 94). When the flaviviruses are categorized based on their preferred host type, we can observe two major clouds (dashed circles) and clusters; viruses that have a vertebrate host and those that have a mosquito-host. Tick-borne viruses largely overlap with the vertebrate host cluster, suggesting that while tick-borne flaviviruses cluster separately, they have the same type of host as mosquito-borne viruses. An interesting exception in the vertebrate cluster is Paraiso Escondido virus, which is located outside the cloud. This may be due to the virus infecting mainly sandflies rather than vertebrates. The centroids were computed based on the multivariate normal distribution of each host type with a confidence level of 0.95. Dimension 1 explains 89.4 percent and dimension 2 contributes to 8.5 percent of the variation.

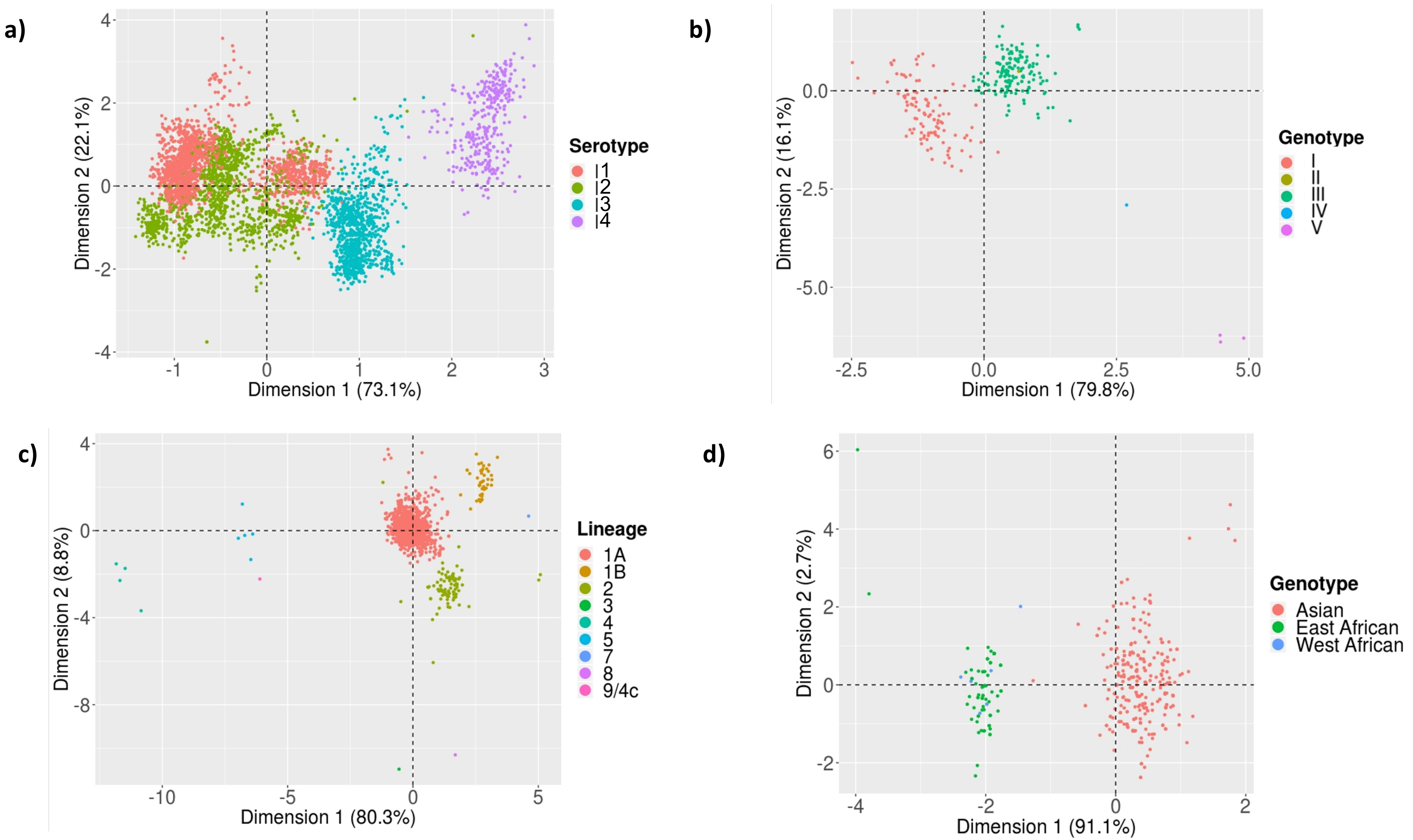

Supplementary figure S3. Intraspecies correspondence analyses of normalized Codon Adaptation Index (nCAI) values of four major mosquito-borne flaviviruses (genus *Flavivirus*). The results show that nCAI is able to discriminate between the different categories of (a) Dengue viruses (N = 4865), (b) Japanese encephalitis viruses (N = 297), (c) West Nile viruses (N = 1619) and Zika viruses (N = 494). Additionally, the distances between separate clusters mirror actual phylogeny. For example, Dengue virus serotypes 1–3, which are clustered close together, are more related to each other than to serotype 4, and the same can be observed with the results of the Zika virus genotypes, in which the Asian and African genotypes cluster separately, but nCAI does not distinguish East and West African genotypes from each other. In panel a, Dimension 1 explains 73.1 percent and Dimension 2 contributes to 22.1 percent of the variability. In panel b, Dimension 1 explains 79.8 percent and Dimension 2 contributes to 16.1 percent of the variability. In panel c, Dimension 1 explains 80.3 percent and Dimension 2 contributes to 8.8 percent of the variability. In panel d, Dimension 1 explains 91.1 percent and Dimension 2 contributes to 2.7 percent of the variability.

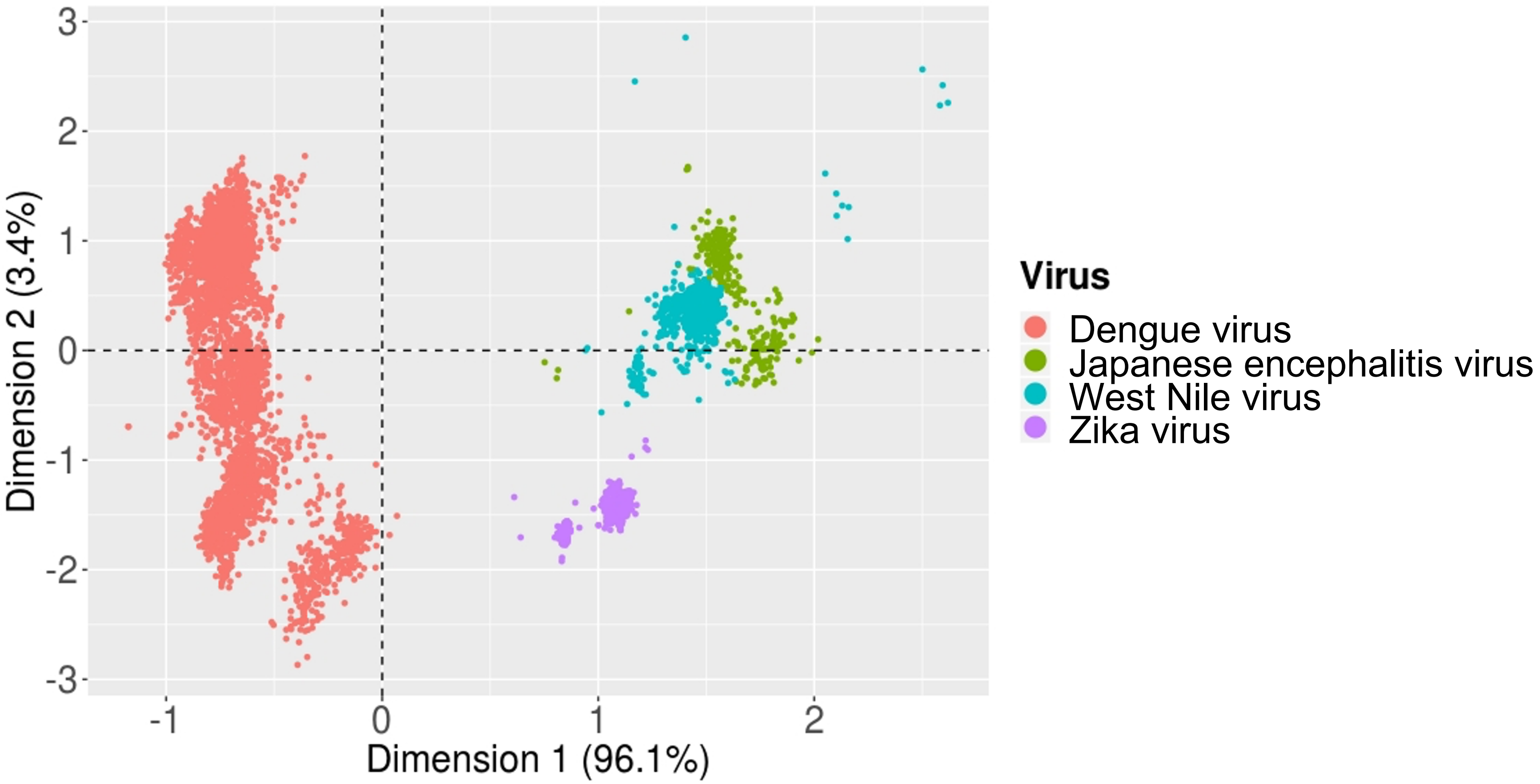

Supplementary figure S4. Interspecies correspondence analysis of normalized Codon Adaptation Index (nCAI) values of four major mosquito-borne flaviviruses, genus *Flavivirus* (N = 7275). The different virus genomes form distinct clusters based on computed nCAI values. The distance between these clusters mirror some phylogenetic relationships among the viruses accurately, e.g. Japanese encephalitis viruses and West Nile viruses share a more recent ancestor than to the other viruses. Of the variation, 96.1 percent is due to Dimension 1 and 3.4 percent is because of Dimension 2.

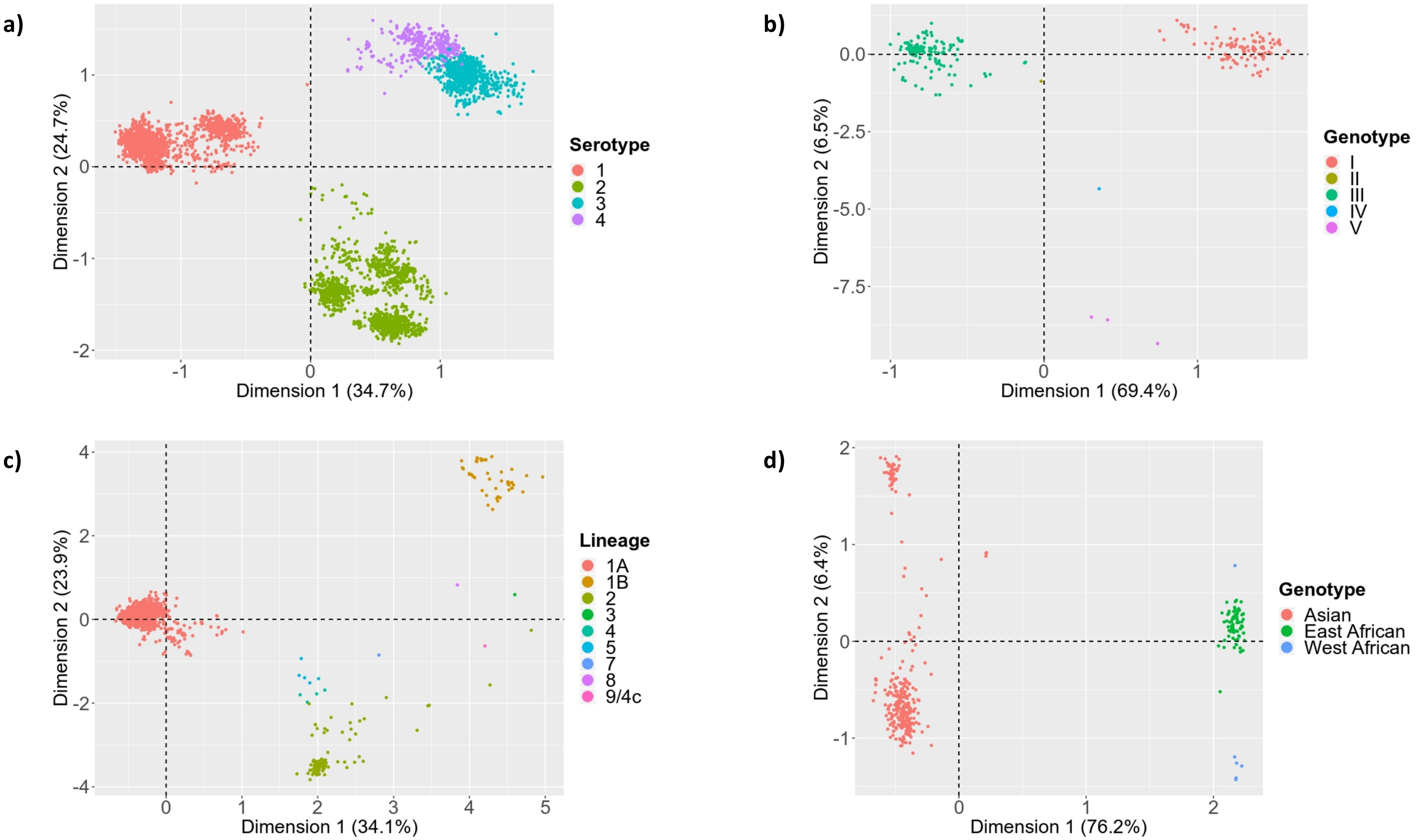

Supplementary figure S5. Intraspecies correspondence analysis of the Relative Synonymous Codon Usage (RSCU) values of major mosquito-borne flaviviruses. With (a) Dengue viruses (N = 4865), (b) Japanese encephalitis viruses (N = 297), (c) West Nile viruses (N = 1619) and Zika viruses (N = 494), different groups of viruses are distinguishable based on their codon usages, although the amount of separation does not necessarily mirror evolutionary relationships with the exception of Zika virus. Each virus outgroup is noted in the legends. Dimension 1 explains 34.7 percent and Dimension 2 attributes to 24.7 percent of the variation in panel a. Dimension 1 explains 69.4 percent and Dimension 2 attributes to 6.5 percent of the variation in panel b. Dimension 1 explains 34.1 percent and Dimension 2 attributes to 23.9 percent of the variation in panel c. Finally, Dimension 1 explains 76.2 percent and Dimension 2 attributes to 6.4 percent of the variation in panel d.

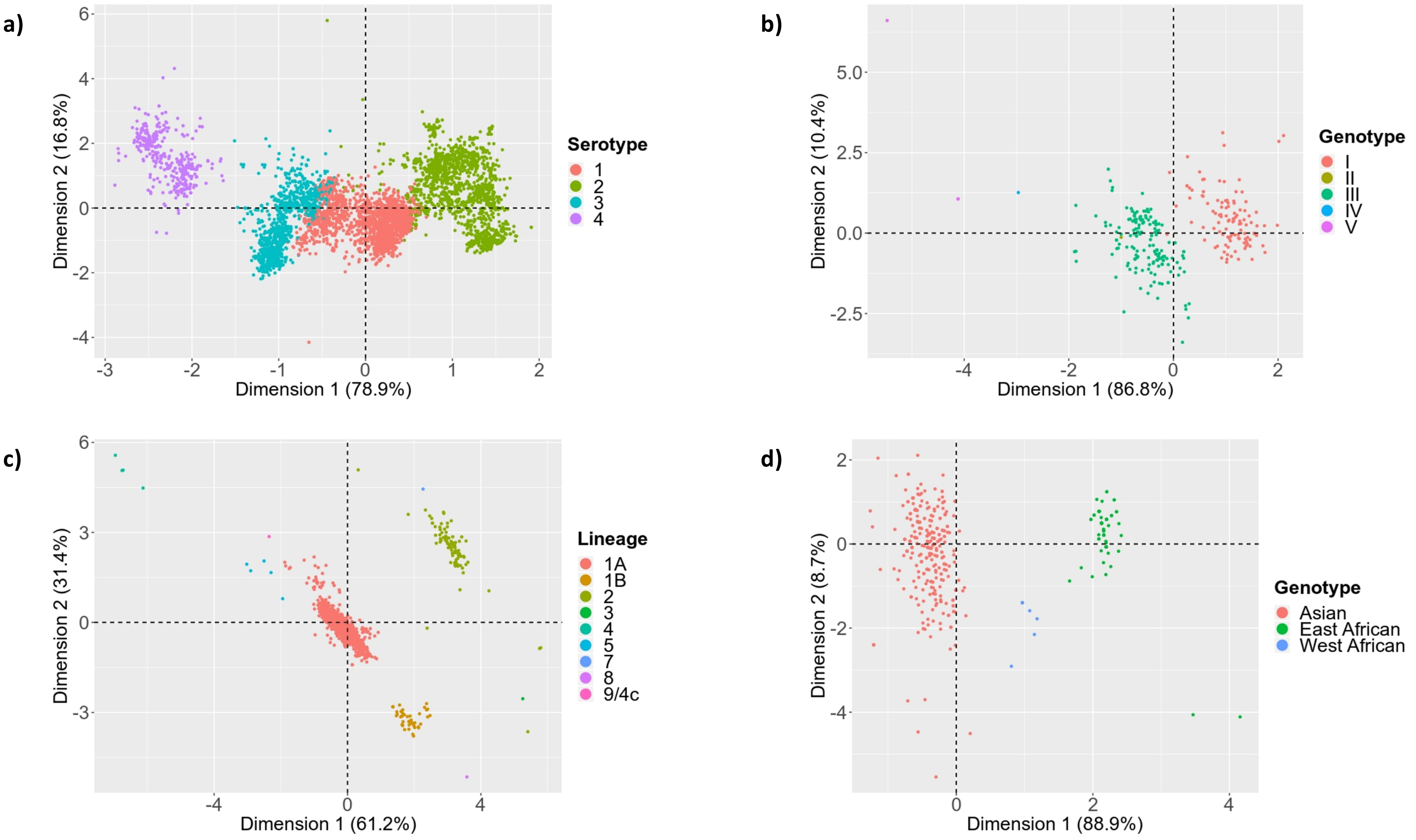

Supplementary figure S6. Intraspecies correspondence analysis of the guanine and cytosine content based on the third nucleotide of each codon (%G3+%C3) of major mosquito-borne flaviviruses. Based on nucleotide compositions, the genomic sequences of (a) Dengue viruses (N = 4865), (b) Japanese encephalitis viruses (N = 297), (c) West Nile viruses (N = 1619) and Zika viruses (N = 494) form distinct cluster that match established categories of each virus. Additionally, the distances between clusters is similar to their phylogenetic relatedness. Each outgroup virus of a respective flavivirus is mentioned in the legend. In panel a, 78.9 percent of the variation is explained by Dimension 1 and 16.8 percent by Dimension 2. In panel b, 86.8 percent of the variation is explained by Dimension 1 and 10.4 percent by Dimension 2. In panel c, 61.2 percent of the variation is explained by Dimension 1 and 31.4 percent by Dimension 2. In panel d, 88.9 percent of the variation is explained by Dimension 1 and 8.7 percent by Dimension 2.

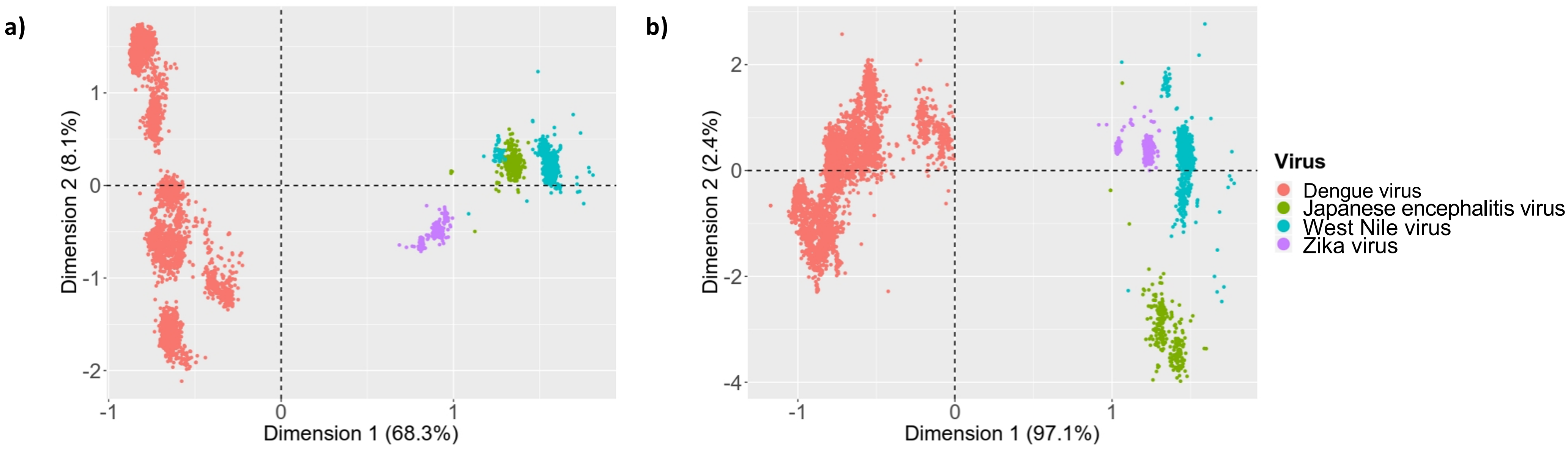

Supplementary figure S7. Interspecies correspondence analysis of the genomic compositions of major mosquito-borne flaviviruses (N = 7275). The viruses form species specific clusters based on their (a) Relative Synonymous Codon Usage (RSCU) values and (b) guanine and cytosine contents based on the third nucleotide of each codon (%G3+%C3). While both methods are capable of distinguishing subgroups within a species, RSCU has a greater ability to separate these groups compared to %G3+%C3. The distances between clusters do not perfectly equate to phylogenetic relationships, e.g. in panel b, Zika viruses are not more related to West Nile viruses than to Dengue viruses. With both analyses, Cell fusing agent virus was used as an outgroup. In panel a, Dimension 1 and 2 explain 68.3 and 8.1 percent of the variation respectively. In panel b, Dimension 1 contributes to 97.1 percent of the variance, while Dimension 2 contributes to 2.4 percent.

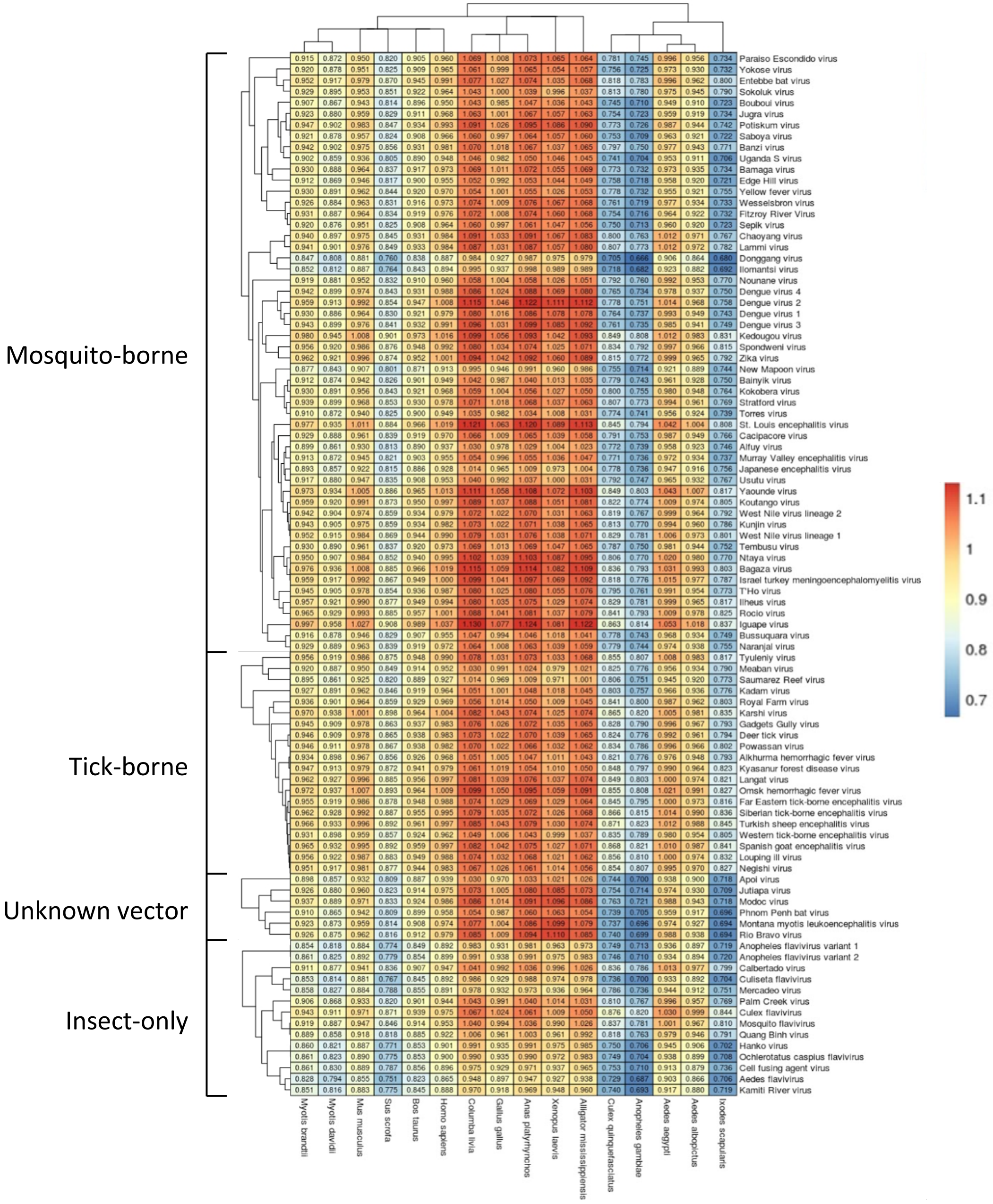

Supplementary figure S8. Normalized Codon Adaptation Index (nCAI) heatmap of flavivirus (genus *Flavivirus*) subgroups (N = 94). The nCAI values show overall over-optimization (nCAI > 1.05) for avians, reptiles and amphibians, and under-optimization for *Culex* and *Anopheles* mosquitoes (nCAI < 0.95). Optimal hosts tend to be mammals and *Aedes* mosquitoes (nCAI 0.95–1.05). The columns are sorted according to taxonomic classification of hosts, and the rows are in accordance with the phylogeny of flaviviruses.

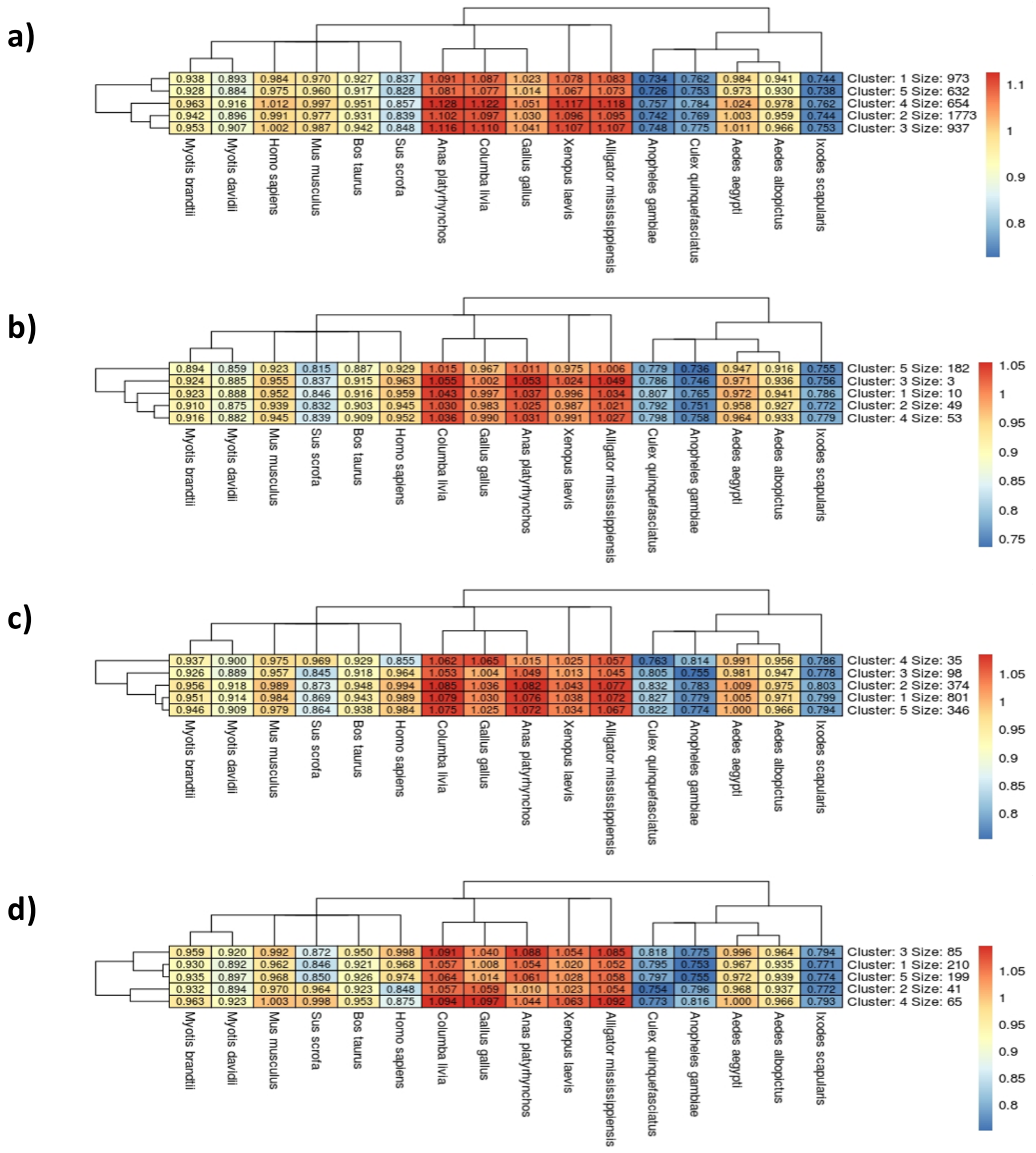

Supplementary figure S9. Intraspecies k-means (5) heatmaps of normalized Codon Adaptation Index (nCAI) values of major mosquito-borne flaviviruses (genus *Flavivirus*). When the nCAI values of (a) Dengue viruses, (b) Japanese encephalitis viruses, (c) West Nile viruses and (d) Zika viruses are plotted in heatmaps, they display similar overall adaptation levels to different host organisms, although there are differences between these viruses. On average the viruses are optimized for mice *(Mus musculus)* and humans *(Homo sapiens),* and *Aedes* mosquitoes, especially *Aedes aegypti* (nCAI 0.95–1.05), thus being likely hosts. The unlikely hosts are the other mammalian hosts, avians, reptiles and amphibians due to over-optimization (nCAI > 1.05), and *Culex* and *Anopheles* mosquitoes, and ticks *(Ixodes scapularis)* due to under-optimization (nCAI < 0.95). There are however exceptions; Japanese encephalitis viruses in panel b have as more likely optimal hosts mostly birds, reptiles and amphibians. The number and size of k-means clusters do not match the current classification of these flaviviruses.
